# Supplementary figures and images for: Novel RNA viruses associated with avian haemosporidian parasites
Source: PLoS One. 2022 Jun 30;17(6):e0269881. doi: 10.1371/journal.pone.0269881 (PMC9246168; doi:10.1371/journal.pone.0269881)

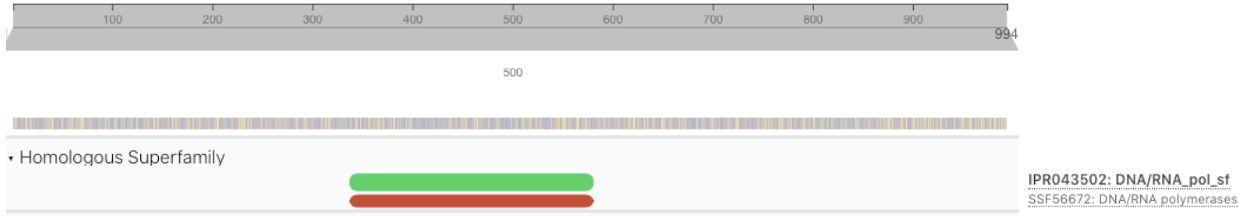

Supplement: S1 Fig — Shows Homology to the DNA/RNA polymerase super family. (TIF) [file pone.0269881.s001.tif]

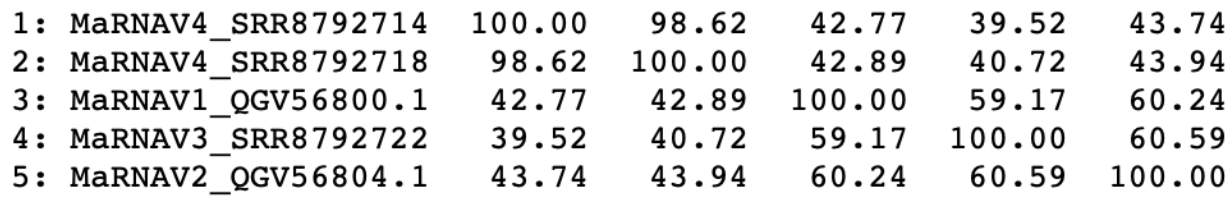

Supplement: S2 Fig — We found that two samples had similar RdRp sequences (~98% amino acid identity), hence we described them both as MaRNAV4. (TIF) [file pone.0269881.s002.tif]
