# Supplementary material for: Novel RNA viruses associated with avian haemosporidian parasites
Source: PLoS One. 2022 Jun 30;17(6):e0269881. doi: 10.1371/journal.pone.0269881 (PMC9246168; doi:10.1371/journal.pone.0269881)
Supplement: S1 File — Results from datasets used to test pipeline used in this study. Results of protein with unknown function including InterPro submission data. (PDF) [file pone.0269881.s003.pdf]

NCBI SRA #	Parasite Infected w/	(+/-) for MaRNAV1	(+/-) for MaRNAV2
SRR7554463	P. *vivax*	+ (98% Identity)	+ (62.1% Identity)
SRR7554428	P. *vivax*	+ (98.6% Identity)	-
SRR7554429	P. *vivax*	+ (97.6% Identity	-
SRR7554464	No Infections	-	-

S1 Table: Data on controls tested through pipeline being used here for virus discovery**.**

Other Protein of Interest	
**SRA number**	SRR8792722
**Host bird Species**	*Acanthis flammea*
**Parasites present**	*Leucocytozoon*
**Length of nt Sequence**	1288nt
**Protein Percent Identity (%)**	28.65% to second segment of MaRNAV1 and 30.45% to MaRNAV2
**Longest ORF**	298aa
**Interpro Results**	MOBIDB_LITE entry, Coil
**Extra Info**	Only found in same sample as MaRNAV3

S2 Table: Gives information about a second protein of interest found in the same sample as MaRNAV3. MaRNAV3 is bi-segmented, with one segment being an RdRp, while the second segment is of unknown function. The protein found above had percent identity to the second segment of MaRNAV3. Percent identity is low so no conclusions could be made
