# Supplementary material for: Novel RNA viruses associated with avian haemosporidian parasites
Source: PLoS One. 2022 Jun 30;17(6):e0269881. doi: 10.1371/journal.pone.0269881 (PMC9246168; doi:10.1371/journal.pone.0269881)
Supplement: S1 Appendix — Results from diamond BLASTx using two databases provided, and an E-value cutoff of 1E-10. Includes Trinity assembly stats report for all transcriptomes used in this study. IQ-Tree files include Newick format tree file and aligned sequences used for analysis. (ZIP) [file pone.0269881.s004.zip › S1Appendix/Phyre2_pdb_files/MaRNAV4_info_tables/ss_report.pdf]

Phyre2

Email	jrodri17@mail.sfsu.edu
Description	MaRNAV4_intense
Date	Wed Feb 2 22:32:35 GMT 2022
Unique Job ID	4a1ebecc6280cf1e

Secondary structure and disorder prediction

1 . . . . 10 . . . . 20 . . . . 30 . . . . 40 . . . . 50 60

Sequence: M S S I I K F T K T D I L R D S A G R V I R S G P W R Y I Y P Q M V R T S P K E Y K Y I L P S G S I L R Y N Y R G K S I

Secondary structure

SS confidence

Disorder: ? ? ? ? ? ? ?

Disorder confidence

. . . . 70 . . . . 80 . . . . 90 . . . . 100 . . . . 110 120

Sequence: L K Q R W E S K E K Y F N D F Q R E G L T K V L S E L G I K T R I I N I I L S R P I H H F L R I E E F V N G L V D A L V

Secondary structure

SS confidence

Disorder: ?

Disorder confidence

. . . . 130 . . . . 140 . . . . 150 . . . . 160 . . . . 170 180

Sequence: C A Y P I I F D R K G K D I R I L R S L Y R K I V N I G T F N L Q L L L K D Y K A F M W N I F H T K A S S V T L E K I F

Secondary structure

SS confidence

Disorder

Disorder confidence

. . . . 190 . . . . 200 . . . . 210 . . . . 220 . . . . 230 240

Sequence: P S N N N I F R I F L K T K M F K N L N K D F K T F Q I L S H L I S S R Q F P Y M G K R T E N Q S L E D F K S V I Q R D

Secondary structure

SS confidence

Disorder: ? ? ? ?

Disorder confidence

. . . . 250 . . . . 260 . . . . 270 . . . . 280 . . . . 290 300

Sequence: Y E P P S E F I E K M F L V G C R I G R I C K R I R P I P I S E N C L H Y S I N S S G E L D H P I S L G A Q A G A V K E

Secondary structure

SS confidence

Disorder: ? ? ? ?

Disorder confidence

. . . . 310 . . . . 320 . . . . 330 . . . . 340 . . . . 350 360

Sequence: V I T S E L T Q L Q D N T F I E D H P V F G K I Q H L E G F Q I W Q Y L F R D N I A I D E F G T P R K V G Y P K D Q P G

Secondary structure

SS confidence

Disorder: ? ? ?

Disorder confidence

. . . . 370 . . . . 380 . . . . 390 . . . . 400 . . . . 410 420

Sequence: R L I G Y D E V L G K Q L F Y L A Y K R I K Q I P I I R V T T V A E M G D K A R I V T V A P W W L G I V E A P I G H L L

Secondary structure

SS confidence

Disorder: ? ? ? ? ? ? ? ? ? ?

Disorder confidence

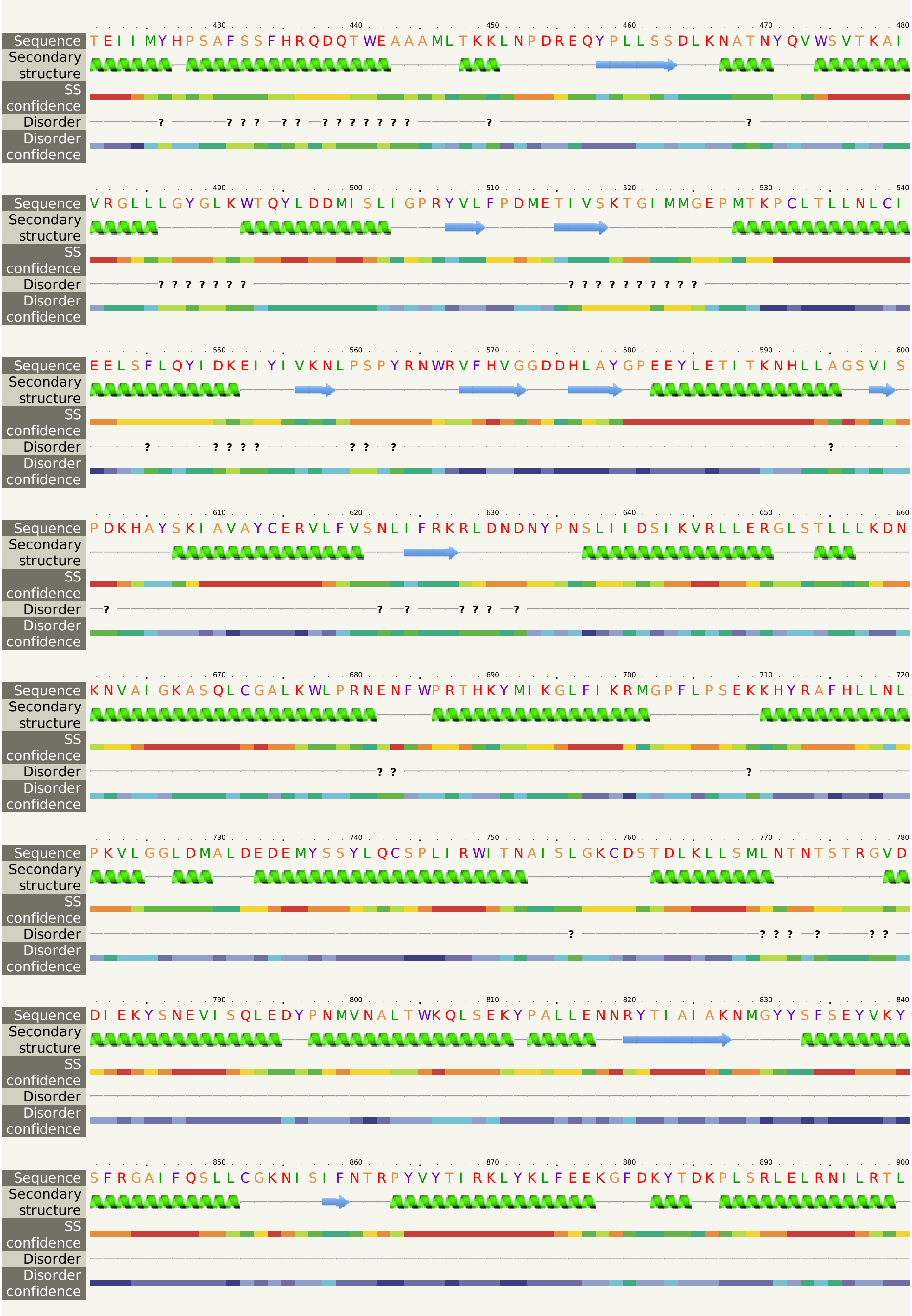
430 440 450 460 470 480
Sequence TEIIMYHPSAFSSFHRQDQTWEAAAMLTKKLNPDREQYPLLSSDLKNATNYQVWSVTKAI
Secondary structure
SS confidence
Disorder
Disorder confidence
490 500 510 520 530 540
Sequence VRGLLLGYGLKWTQYLDDMISLIGPRYVLFPDMETIVSKTGIMMGEPMTKPCLTLLNLCI
550 560 570 580 590 600
Sequence EELSFLQYIDKEIYIVKNLPSPYRNWRVFHVGGDDHLAYGPEEYLETITKNHLLAGSVIS
610 620 630 640 650 660
Sequence PDKHAYSKIAVAYCERVLFVSNLIFRKRLDNDNYPNSLIIDSIKVRLLERGLSTLLLKDN
670 680 690 700 710 720
Sequence KNVAIGKASQLCGALKWLPRNENFWPRTHKYMIKGLFIKRMGPFLPSEKKHYRAFHLLNL
730 740 750 760 770 780
Sequence PKVLGGLDMALDEDEMYSSYLQCSPLIRWITNAISLGKCDSTDLKLLSMLNTNTSTRGVD
790 800 810 820 830 840
Sequence DIEKYSNEVISQLEDYPNMVNALTWKQLSEKYPALLENNRYTIAIAKNMGYYSFSEYVKY
850 860 870 880 890 900
Sequence SFRGAIFQSLLCGKNISIFNTRPYVYTIRKLYKLFEEKGFDKYTDKPLSRLELRNILRTL

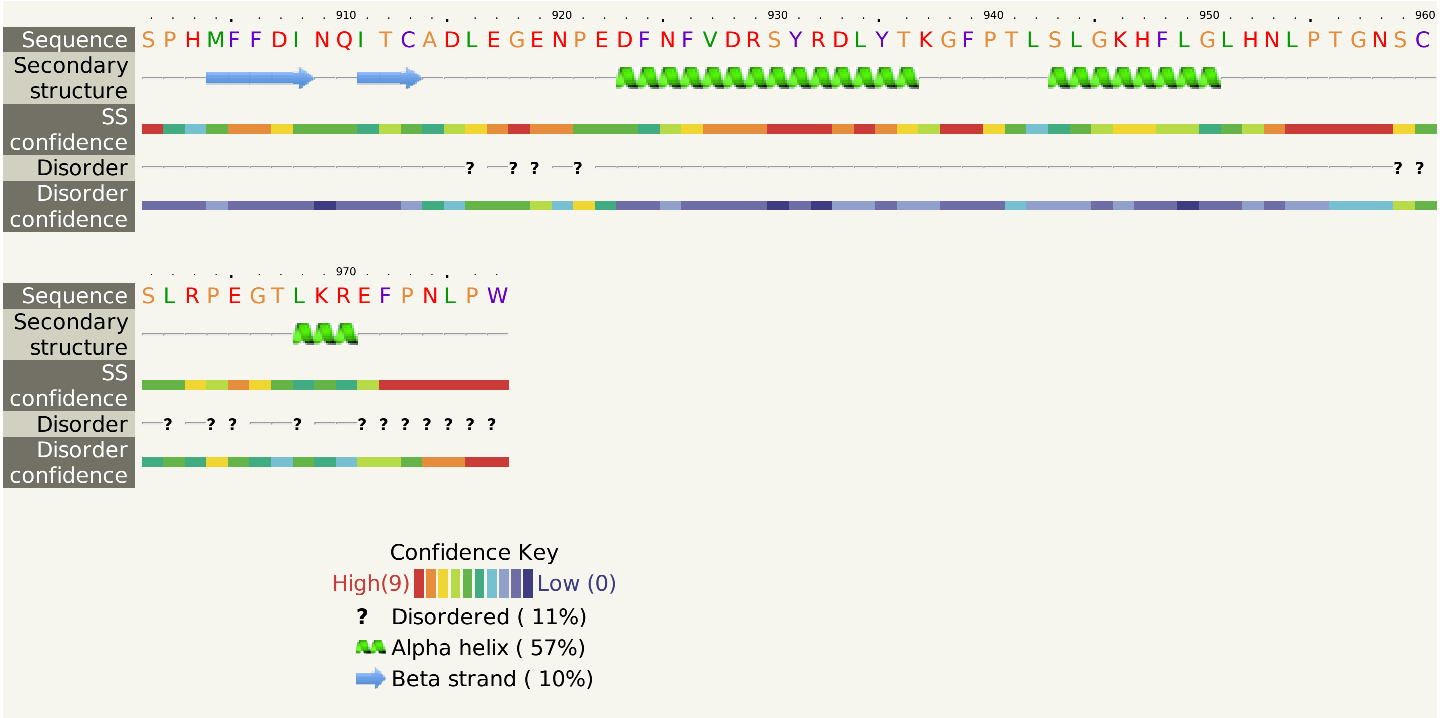
910
920
930
940
950
960
Sequence
SPHMFFDINQITCADLEGENPEDFNFVDRSYRDLYTKGFPTLSLGKHFLGLHNLPTGNSC
Secondary structure
SS confidence
Disorder
Disorder confidence
970
Sequence
SLRPEGTLKREFPNLPW
Secondary structure
SS confidence
Disorder
Disorder confidence
Confidence Key
High(9)
Low (0)
Disordered (11%)
Alpha helix (57%)
Beta strand (10%)
