# Supplementary material for: Novel RNA viruses associated with avian haemosporidian parasites
Source: PLoS One. 2022 Jun 30;17(6):e0269881. doi: 10.1371/journal.pone.0269881 (PMC9246168; doi:10.1371/journal.pone.0269881)
Supplement: S1 Appendix — Results from diamond BLASTx using two databases provided, and an E-value cutoff of 1E-10. Includes Trinity assembly stats report for all transcriptomes used in this study. IQ-Tree files include Newick format tree file and aligned sequences used for analysis. (ZIP) [file pone.0269881.s004.zip › S1Appendix/Phyre2_pdb_files/MaRNAV4_info_tables/hit_report.pdf]

Phyre2

Email	jrodri17@mail.sfsu.edu
Description	MaRNAV4_intense
Date	Wed Feb 2 22:32:35 GMT 2022
Unique Job ID	4a1ebecc6280cf1e

Detailed template information

#	Template	Alignment Coverage	3D Model	Confidence	% i.d.	Template Information
1	d1u09a_	Alignment	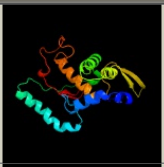	93.1	16	**Fold:** DNA/RNA polymerases **Superfamily:** DNA/RNA polymerases **Family:** RNA-dependent RNA-polymerase **PDB entry:** PDBe RCSB PDBj
2	c2uutA_	Alignment	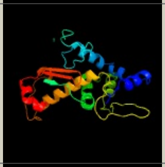	89.6	14	**PDB header:**hydrolase **Chain:** A: **PDB Molecule:**rna-directed rna polymerase; **PDBTitle:** the 2.4 angstrom resolution structure of the d346g mutant of the2 sapporo virus rdrp polymerase **PDB Entry:** PDBe RCSB PDBj
3	c2b43D_	Alignment	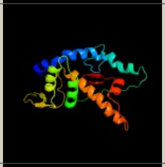	89.3	15	**PDB header:**viral protein **Chain:** D: **PDB Molecule:**non-structural polyprotein; **PDBTitle:** crystal structure of the norwalk virus rna dependent rna polymerase2 from strain hu/nlv/dresden174/1997/ge **PDB Entry:** PDBe RCSB PDBj
4	c5y6rA_	Alignment	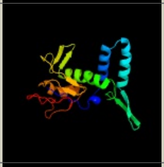	86.9	21	**PDB header:**transferase **Chain:** A: **PDB Molecule:**genome polyprotein; **PDBTitle:** crystal structure of csfv ns5b **PDB Entry:** PDBe RCSB PDBj
5	c3nahC_	Alignment	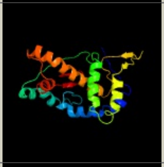	86.8	13	**PDB header:**transferase **Chain:** C: **PDB Molecule:**rna dependent rna polymerase; **PDBTitle:** crystal structures and functional analysis of murine norovirus rna-2 dependent rna polymerase **PDB Entry:** PDBe RCSB PDBj
6	d1khva_	Alignment	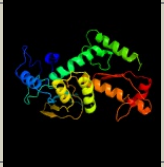	85.8	15	**Fold:** DNA/RNA polymerases **Superfamily:** DNA/RNA polymerases **Family:** RNA-dependent RNA-polymerase **PDB entry:** PDBe RCSB PDBj
7	c4nz0F_	Alignment	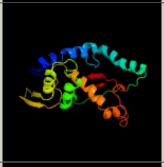	83.1	19	**PDB header:**transferase **Chain:** F: **PDB Molecule:**genome polyprotein; **PDBTitle:** the emcv 3dpol structure at 2.8a resolution **PDB Entry:** PDBe RCSB PDBj
8	d1sh0a_	Alignment	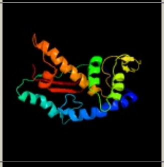	79.1	16	**Fold:** DNA/RNA polymerases **Superfamily:** DNA/RNA polymerases **Family:** RNA-dependent RNA-polymerase **PDB entry:** PDBe RCSB PDBj
9	c3n6mA_	Alignment	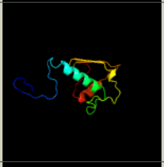	73.7	22	**PDB header:**transferase **Chain:** A: **PDB Molecule:**rna-dependent rna polymerase; **PDBTitle:** crystal structure of ev71 rdrp in complex with gtp **PDB Entry:** PDBe RCSB PDBj
10	d1s48a_	Alignment	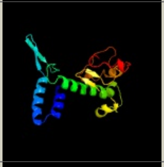	59.3	20	**Fold:** DNA/RNA polymerases **Superfamily:** DNA/RNA polymerases **Family:** RNA-dependent RNA-polymerase **PDB entry:** PDBe RCSB PDBj
11	d1xr7a_	Alignment	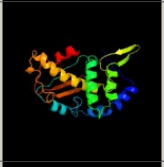	55.7	18	**Fold:** DNA/RNA polymerases **Superfamily:** DNA/RNA polymerases **Family:** RNA-dependent RNA-polymerase **PDB entry:** PDBe RCSB PDBj

12	c5i62A_	Alignment	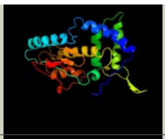	54.1	18	**PDB header:** viral protein, replication<br>**Chain:** A: **PDB Molecule:** potential rna-dependent rna polymerase;<br>**PDBTitle:** crystal structure of the insertion loop deletion mutant of the rna-2 dependent rna polymerase of a human picorbirnavirus<br>**PDB Entry:** PDBe RCSB PDBj
13	d1xr6a_	Alignment	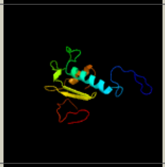	52.6	17	**Fold:** DNA/RNA polymerases<br>**Superfamily:** DNA/RNA polymerases<br>**Family:** RNA-dependent RNA-polymerase<br>**PDB entry:** PDBe RCSB PDBj
14	c6r1iB_	Alignment	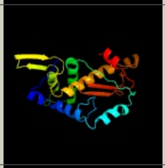	52.4	17	**PDB header:** transferase<br>**Chain:** B: **PDB Molecule:** genome polyprotein;<br>**PDBTitle:** structure of porcine aichi virus polymerase<br>**PDB Entry:** PDBe RCSB PDBj
15	c2yqyB_	Alignment	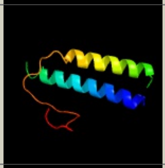	51.2	25	**PDB header:** structural genomics, unknown function<br>**Chain:** B: **PDB Molecule:** hypothetical protein ttha0303;<br>**PDBTitle:** crystal structure of tt2238, a four-helix bundle protein<br>**PDB Entry:** PDBe RCSB PDBj
16	c2ijd1_	Alignment	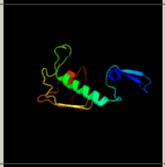	48.6	20	**PDB header:** hydrolase, transferase<br>**Chain:** 1: **PDB Molecule:** picornain 3c, rna-directed rna polymerase;<br>**PDBTitle:** crystal structure of the poliovirus precursor protein 3cd<br>**PDB Entry:** PDBe RCSB PDBj
17	d1j9ba_	Alignment	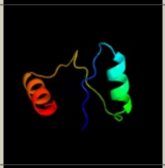	42.8	29	**Fold:** Thioredoxin fold<br>**Superfamily:** Thioredoxin-like<br>**Family:** ArsC-like<br>**PDB entry:** PDBe RCSB PDBj
18	d1ra6a_	Alignment	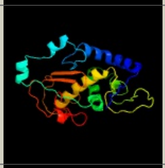	41.9	15	**Fold:** DNA/RNA polymerases<br>**Superfamily:** DNA/RNA polymerases<br>**Family:** RNA-dependent RNA-polymerase<br>**PDB entry:** PDBe RCSB PDBj
19	d1nlna_	Alignment	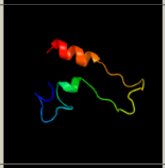	31.7	21	**Fold:** Cysteine proteinases<br>**Superfamily:** Cysteine proteinases<br>**Family:** Adenain-like<br>**PDB entry:** PDBe RCSB PDBj
20	c6j4uA_	Alignment	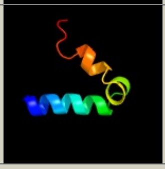	27.6	32	**PDB header:** hydrolase<br>**Chain:** A: **PDB Molecule:** tubulinyl-tyr carboxypeptidase 1;<br>**PDBTitle:** structural basis of tubulin detyrosination by vasohibins-svbp enzyme2 complex and functional implications<br>**PDB Entry:** PDBe RCSB PDBj
21	c6lcuA_	Alignment	not modelled	25.4	30	**PDB header:** isomerase<br>**Chain:** A: **PDB Molecule:** mtsase;<br>**PDBTitle:** structure of maltooligosyltrehalose synthase from arthrobacter ramosus<br>**PDB Entry:** PDBe RCSB PDBj
22	d1xr5a_	Alignment	not modelled	23.6	15	**Fold:** DNA/RNA polymerases<br>**Superfamily:** DNA/RNA polymerases<br>**Family:** RNA-dependent RNA-polymerase<br>**PDB entry:** PDBe RCSB PDBj
23	c5o60U_	Alignment	not modelled	20.9	26	**PDB header:** ribosome<br>**Chain:** U: **PDB Molecule:** 50s ribosomal protein l23;<br>**PDBTitle:** structure of the 50s large ribosomal subunit from mycobacterium2 smegmatis<br>**PDB Entry:** PDBe RCSB PDBj
24	c4v194_	Alignment	not modelled	18.8	27	**PDB header:** ribosome<br>**Chain:** 4: **PDB Molecule:** mitoribosomal protein bl31m, mrpl55;<br>**PDBTitle:** structure of the large subunit of the mammalian mitoribosome, part 12 of 2<br>**PDB Entry:** PDBe RCSB PDBj
25	c6qbyA_	Alignment	not modelled	17.6	26	**PDB header:** cytosolic protein<br>**Chain:** A: **PDB Molecule:** tubulinyl-tyr carboxypeptidase 2;<br>**PDBTitle:** crystal structure of vash 2 in complex with svbp<br>**PDB Entry:** PDBe RCSB PDBj
26	d2nn6c1	Alignment	not modelled	16.7	20	**Fold:** Ribosomal protein S5 domain 2-like<br>**Superfamily:** Ribosomal protein S5 domain 2-like<br>**Family:** Ribonuclease PH domain 1-like<br>**PDB entry:** PDBe RCSB PDBj
27	c6wj6E_	Alignment	not modelled	16.3	57	**PDB header:** photosynthesis<br>**Chain:** E: **PDB Molecule:** cytochrome b559 subunit alpha;<br>**PDBTitle:** cryo-em structure of apo-photosystem ii from synechocystis sp. pcc2 6803<br>**PDB Entry:** PDBe RCSB PDBj
						**PDB header:** ribosome<br>**Chain:** M: **PDB Molecule:**

28	c4v1am_	Alignment	not modelled	16.1	24	**PDBTitle:** structure of the large subunit of the mammalian mitoribosome, part 22 of 2<br>**PDB Entry:** PDBe RCSB PDBj
29	c3kziE_	Alignment	not modelled	16.1	53	**PDB header:**electron transport<br>**Chain:** E: **PDB Molecule:**cytochrome b559 subunit alpha;<br>**PDBTitle:** crystal structure of monomeric form of cyanobacterial photosystem ii<br>**PDB Entry:** PDBe RCSB PDBj
30	c3fvyA_	Alignment	not modelled	15.3	21	**PDB header:**hydrolase<br>**Chain:** A: **PDB Molecule:**dipeptidyl-peptidase 3;<br>**PDBTitle:** crystal structure of human dipeptidyl peptidase iii<br>**PDB Entry:** PDBe RCSB PDBj
31	c3ebwA_	Alignment	not modelled	15.3	15	**PDB header:**allergen<br>**Chain:** A: **PDB Molecule:**per a 4 allergen;<br>**PDBTitle:** crystal structure of major allergens, per a 4 from cockroaches<br>**PDB Entry:** PDBe RCSB PDBj
32	c2mu0A_	Alignment	not modelled	14.8	28	**PDB header:**oxidoreductase<br>**Chain:** A: **PDB Molecule:**arsenate reductase;<br>**PDBTitle:** solution structure of a putative arsenate reductase from brucella2 melitensis. seattle structural genomics center for infectious disease3 target braba.00073.a<br>**PDB Entry:** PDBe RCSB PDBj
33	d2axte1	Alignment	not modelled	14.7	57	**Fold:** Single transmembrane helix<br>**Superfamily:** Cytochrome b559 subunits<br>**Family:** Cytochrome b559 subunits<br>**PDB entry:** PDBe RCSB PDBj
34	d2j01x1	Alignment	not modelled	14.3	26	**Fold:** Ribosomal proteins S24e, L23 and L15e<br>**Superfamily:** Ribosomal proteins S24e, L23 and L15e<br>**Family:** L23p<br>**PDB entry:** PDBe RCSB PDBj
35	d2zjrq1	Alignment	not modelled	14.1	17	**Fold:** Ribosomal proteins S24e, L23 and L15e<br>**Superfamily:** Ribosomal proteins S24e, L23 and L15e<br>**Family:** L23p<br>**PDB entry:** PDBe RCSB PDBj
36	c1deqO_	Alignment	not modelled	13.5	27	**PDB header:**blood clotting<br>**Chain:** O: **PDB Molecule:**fibrinogen (beta chain);<br>**PDBTitle:** the crystal structure of modified bovine fibrinogen (at ~42 angstrom resolution)<br>**PDB Entry:** PDBe RCSB PDBj
37	c3j3vT_	Alignment	not modelled	13.3	17	**PDB header:**ribosome<br>**Chain:** T: **PDB Molecule:**50s ribosomal protein l23;<br>**PDBTitle:** atomic model of the immature 50s subunit from bacillus subtilis (state2 i-a)<br>**PDB Entry:** PDBe RCSB PDBj
38	c7lf7M_	Alignment	not modelled	13.3	50	**PDB header:**immune system<br>**Chain:** M: **PDB Molecule:**apolipoprotein l1;<br>**PDBTitle:** fab 6d12 bound to apol1 ntd<br>**PDB Entry:** PDBe RCSB PDBj
39	c2cjqA_	Alignment	not modelled	13.0	26	**PDB header:**transferase<br>**Chain:** A: **PDB Molecule:**rna-directed rna polymerase;<br>**PDBTitle:** bovine viral diarrhea virus cp7-r12 rna-dependent rna2 polymerase<br>**PDB Entry:** PDBe RCSB PDBj
40	d2hf5a1	Alignment	not modelled	12.8	38	**Fold:** EF Hand-like<br>**Superfamily:** EF-hand<br>**Family:** Calmodulin-like<br>**PDB entry:** PDBe RCSB PDBj
41	d1kx9b_	Alignment	not modelled	12.7	18	**Fold:** alpha-alpha superhelix<br>**Superfamily:** Chemosensory protein Csp2<br>**Family:** Chemosensory protein Csp2<br>**PDB entry:** PDBe RCSB PDBj
42	c3jcue_	Alignment	not modelled	12.5	50	**PDB header:**membrane protein<br>**Chain:** E: **PDB Molecule:**cytochrome b559 subunit alpha;<br>**PDBTitle:** cryo-em structure of spinach psii-lhcii supercomplex at 3.2 angstrom2 resolution<br>**PDB Entry:** PDBe RCSB PDBj
43	c3mtuE_	Alignment	not modelled	12.4	26	**PDB header:**contractile protein<br>**Chain:** E: **PDB Molecule:**capsid assembly scaffolding protein,tropomyosin alpha-1<br>**PDBTitle:** structure of the tropomyosin overlap complex from chicken smooth2 muscle<br>**PDB Entry:** PDBe RCSB PDBj
44	c3kzwD_	Alignment	not modelled	12.2	24	**PDB header:**hydrolase<br>**Chain:** D: **PDB Molecule:**cytosol aminopeptidase;<br>**PDBTitle:** crystal structure of cytosol aminopeptidase from staphylococcus aureus2 col<br>**PDB Entry:** PDBe RCSB PDBj
45	d1g3nc2	Alignment	not modelled	12.0	38	**Fold:** Cyclin-like<br>**Superfamily:** Cyclin-like<br>**Family:** Cyclin<br>**PDB entry:** PDBe RCSB PDBj
46	c4wfbQ_	Alignment	not modelled	11.9	17	**PDB header:**ribosome<br>**Chain:** Q: **PDB Molecule:**50s ribosomal protein l23;<br>**PDBTitle:** the crystal structure of the large ribosomal subunit of staphylococcus2 aureus in complex with bc-3205<br>**PDB Entry:** PDBe RCSB PDBj
47	c6p7vC_	Alignment	not modelled	11.7	19	**PDB header:**dna binding protein<br>**Chain:** C: **PDB Molecule:**ctf13;<br>**PDBTitle:** structure of the k. lactis cbf3 core<br>**PDB Entry:** PDBe RCSB PDBj
48	d1u0sy_	Alignment	not modelled	11.5	20	**Fold:** Flavodoxin-like<br>**Superfamily:** CheY-like<br>**Family:** CheY-related<br>**PDB entry:** PDBe RCSB PDBj
49	c5c6kB_	Alignment	not modelled	11.3	11	**PDB header:**hydrolase<br>**Chain:** B: **PDB Molecule:**integrase;<br>**PDBTitle:** bacteriophage p2 integrase catalytic domain<br>**PDB Entry:** PDBe RCSB PDBj
50	c6p7wC_	Alignment	not modelled	10.9	19	**PDB header:**dna binding protein<br>**Chain:** C: **PDB Molecule:**ctf13;<br>**PDBTitle:** structure of the k. lactis cbf3 core - ndc10 d1 complex<br>**PDB Entry:** PDBe RCSB PDBj

51	c6swgC_	Alignment	not modelled	10.9	43	**PDB header:**gene regulation<br>**Chain:** C: **PDB Molecule:**protein tasor;<br>**PDBTitle:** crystal structure of the tasor-periphilin core complex<br>**PDB Entry:** PDBe RCSB PDBj
52	d2r6gf1	Alignment	not modelled	10.8	46	**Fold:** MalF N-terminal region-like<br>**Superfamily:** MalF N-terminal region-like<br>**Family:** MalF N-terminal region-like<br>**PDB entry:** PDBe RCSB PDBj
53	c3b42B_	Alignment	not modelled	10.5	38	**PDB header:**signaling protein<br>**Chain:** B: **PDB Molecule:**methyl-accepting chemotaxis protein, putative;<br>**PDBTitle:** periplasmic sensor domain of chemotaxis protein gsu0935<br>**PDB Entry:** PDBe RCSB PDBj
54	c6wu9U_	Alignment	not modelled	9.8	17	**PDB header:**ribosome<br>**Chain:** U: **PDB Molecule:**50s ribosomal protein l23;<br>**PDBTitle:** 50s subunit of 70s ribosome enterococcus faecalis multibody refinement<br>**PDB Entry:** PDBe RCSB PDBj
55	c6dzpg_	Alignment	not modelled	9.5	25	**PDB header:**ribosome<br>**Chain:** G: **PDB Molecule:**50s ribosomal protein l6;<br>**PDBTitle:** cryo-em structure of mycobacterium smegmatis c(minus) 50s ribosomal2 subunit<br>**PDB Entry:** PDBe RCSB PDBj
56	c6y6kA_	Alignment	not modelled	9.3	19	**PDB header:**viral protein<br>**Chain:** A: **PDB Molecule:**rna-dependent rna polymerase;<br>**PDBTitle:** cryo-em structure of a phenuiviridae l protein<br>**PDB Entry:** PDBe RCSB PDBj
57	c7lfaC_	Alignment	not modelled	9.3	48	**PDB header:**immune system<br>**Chain:** C: **PDB Molecule:**apolipoprotein l1;<br>**PDBTitle:** fab 3b6 bound to apol1 ntd<br>**PDB Entry:** PDBe RCSB PDBj
58	c2wb6A_	Alignment	not modelled	9.3	53	**PDB header:**viral protein<br>**Chain:** A: **PDB Molecule:**afv1-102;<br>**PDBTitle:** crystal structure of afv1-102, a protein from the acidianus2 filamentous virus 1<br>**PDB Entry:** PDBe RCSB PDBj
59	c4dtfA_	Alignment	not modelled	9.3	39	**PDB header:**toxin<br>**Chain:** A: **PDB Molecule:**vgrg protein;<br>**PDBTitle:** structure of a vgrg vibrio cholerae toxin acd domain in complex with2 amp-pnp and mg++<br>**PDB Entry:** PDBe RCSB PDBj
60	d1dp7p_	Alignment	not modelled	9.1	28	**Fold:** DNA/RNA-binding 3-helical bundle<br>**Superfamily:** "Winged helix" DNA-binding domain<br>**Family:** P4 origin-binding domain-like<br>**PDB entry:** PDBe RCSB PDBj
61	c7apkJ_	Alignment	not modelled	9.0	16	**PDB header:**gene regulation<br>**Chain:** J: **PDB Molecule:**tho complex subunit 2;<br>**PDBTitle:** structure of the human tho - uap56 complex<br>**PDB Entry:** PDBe RCSB PDBj
62	c5n0lC_	Alignment	not modelled	8.6	30	**PDB header:**transcription<br>**Chain:** C: **PDB Molecule:**gtp-sensing transcriptional pleiotropic repressor cody;<br>**PDBTitle:** the structure of the cofactor binding gaf domain of the nutrient2 sensor cody from clostridium difficile<br>**PDB Entry:** PDBe RCSB PDBj
63	c2hbzA_	Alignment	not modelled	8.4	18	**PDB header:**hydrolase/hydrolase inhibitor<br>**Chain:** A: **PDB Molecule:**caspase-1;<br>**PDBTitle:** crystal structure of human caspase-1 (arg286->ala, glu390->ala) in2 complex with 3-[2-(2-benzyloxycarbonylamino-3-methyl-butyrylamino)-3 propionylamino]-4-oxo-pentanoic acid (z-vad-fmk)<br>**PDB Entry:** PDBe RCSB PDBj
64	c7luvC_	Alignment	not modelled	8.3	19	**PDB header:**rna binding protein<br>**Chain:** C: **PDB Molecule:**tho complex subunit 2;<br>**PDBTitle:** cryo-em structure of the yeast tho-sub2 complex<br>**PDB Entry:** PDBe RCSB PDBj
65	c4cmqB_	Alignment	not modelled	8.2	41	**PDB header:**hydrolase<br>**Chain:** B: **PDB Molecule:**crispr-associated endonuclease cas9/csn1;<br>**PDBTitle:** crystal structure of mn-bound s.pyogenes cas9<br>**PDB Entry:** PDBe RCSB PDBj
66	c2mbhB_	Alignment	not modelled	8.0	40	**PDB header:**transcription<br>**Chain:** B: **PDB Molecule:**krueppel-like factor 1;<br>**PDBTitle:** nmr structure of eklf(22-40)/ubiquitin complex<br>**PDB Entry:** PDBe RCSB PDBj
67	c3a9fA_	Alignment	not modelled	7.8	14	**PDB header:**electron transport<br>**Chain:** A: **PDB Molecule:**cytochrome c;<br>**PDBTitle:** crystal structure of the c-terminal domain of cytochrome cz from2 chlorobium tepidum<br>**PDB Entry:** PDBe RCSB PDBj
68	c6s5wA_	Alignment	not modelled	7.8	24	**PDB header:**structural protein<br>**Chain:** A: **PDB Molecule:**surface protein;<br>**PDBTitle:** structure of rib domain 'rib long' from lactobacillus acidophilus<br>**PDB Entry:** PDBe RCSB PDBj
69	c2rhbD_	Alignment	not modelled	7.7	34	**PDB header:**viral protein<br>**Chain:** D: **PDB Molecule:**uridylate-specific endoribonuclease;<br>**PDBTitle:** crystal structure of nsp15-h234a mutant- hexamer in asymmetric unit<br>**PDB Entry:** PDBe RCSB PDBj
70	c2kncA_	Alignment	not modelled	7.6	26	**PDB header:**cell adhesion<br>**Chain:** A: **PDB Molecule:**integrin alpha-iib;<br>**PDBTitle:** platelet integrin alfaiib-beta3 transmembrane-cytoplasmic2 heterocomplex<br>**PDB Entry:** PDBe RCSB PDBj
71	c6k4fU_	Alignment	not modelled	7.5	22	**PDB header:**biosynthetic protein<br>**Chain:** U: **PDB Molecule:**duf1987 domain-containing protein;<br>**PDBTitle:** siac of pseudomonas aeruginosa<br>**PDB Entry:** PDBe RCSB PDBj
72	c5nvkF_	Alignment	not modelled	7.4	46	**PDB header:**translation<br>**Chain:** F: **PDB Molecule:**grb10-interacting gyf protein 1;<br>**PDBTitle:** crystal structure of the human 4ehp-gigyf1 complex<br>**PDB Entry:** PDBe RCSB PDBj

73	d1n8va_	Alignment	not modelled	7.3	18	**Fold:** alpha-alpha superhelix<br>**Superfamily:** Chemosensory protein Csp2<br>**Family:** Chemosensory protein Csp2<br>**PDB entry:** PDBe RCSB PDBj
74	c1pqrA_	Alignment	not modelled	7.2	60	**PDB header:**toxin<br>**Chain:** A: **PDB Molecule:**alpha-a-conotoxin eiva;<br>**PDBTitle:** solution conformation of alphaa-conotoxin eiva<br>**PDB Entry:** PDBe RCSB PDBj
75	c2xpnB_	Alignment	not modelled	7.1	83	**PDB header:**transcription<br>**Chain:** B: **PDB Molecule:**chromatin structure modulator;<br>**PDBTitle:** crystal structure of a spt6-iws1(spn1) complex from2 encephalitozoon cuniculi, form i<br>**PDB Entry:** PDBe RCSB PDBj
76	c5t7aA_	Alignment	not modelled	6.9	16	**PDB header:**sugar binding protein<br>**Chain:** A: **PDB Molecule:**bh0236 protein;<br>**PDBTitle:** crystal structure of br derivative bhcbm56<br>**PDB Entry:** PDBe RCSB PDBj
77	c4bjiA_	Alignment	not modelled	6.8	25	**PDB header:**dna binding protein<br>**Chain:** A: **PDB Molecule:**transcription factor tau subunit sfc1;<br>**PDBTitle:** sfc1-dbd<br>**PDB Entry:** PDBe RCSB PDBj
78	c6dt0D_	Alignment	not modelled	6.8	44	**PDB header:**transport protein<br>**Chain:** D: **PDB Molecule:**mitochondrial calcium uniporter;<br>**PDBTitle:** cryo-em structure of a mitochondrial calcium uniporter<br>**PDB Entry:** PDBe RCSB PDBj
79	c2rr7A_	Alignment	not modelled	6.8	14	**PDB header:**motor protein<br>**Chain:** A: **PDB Molecule:**dynein heavy chain 9;<br>**PDBTitle:** microtubule binding domain of dynein-c<br>**PDB Entry:** PDBe RCSB PDBj
80	c2ys5A_	Alignment	not modelled	6.8	19	**PDB header:**signaling protein<br>**Chain:** A: **PDB Molecule:**fibroblast growth factor receptor substrate 3;<br>**PDBTitle:** solution structure of the complex of the ptb domain of snt-2 and 19-2 residue peptide (aa 1571-1589) of halk<br>**PDB Entry:** PDBe RCSB PDBj
81	c6fe8D_	Alignment	not modelled	6.8	35	**PDB header:**dna binding protein<br>**Chain:** D: **PDB Molecule:**centromere dna-binding protein complex cbf3 subunit c;<br>**PDBTitle:** cryo-em structure of the core centromere binding factor 3 complex<br>**PDB Entry:** PDBe RCSB PDBj
82	c6kmtC_	Alignment	not modelled	6.7	18	**PDB header:**immune system<br>**Chain:** C: **PDB Molecule:**caspase-4;<br>**PDBTitle:** p32 of caspase-11 mutant c254a<br>**PDB Entry:** PDBe RCSB PDBj
83	c1deqF_	Alignment	not modelled	6.6	35	**PDB header:**blood clotting<br>**Chain:** F: **PDB Molecule:**fibrinogen (gamma chain);<br>**PDBTitle:** the crystal structure of modified bovine fibrinogen (at ~42 angstrom resolution)<br>**PDB Entry:** PDBe RCSB PDBj
84	c4xxfA_	Alignment	not modelled	6.3	21	**PDB header:**lyase<br>**Chain:** A: **PDB Molecule:**fuculose-1-phosphate aldolase;<br>**PDBTitle:** l-fuculose 1-phosphate aldolase from glaciozyma antarctica pi12<br>**PDB Entry:** PDBe RCSB PDBj
85	c2l5bA_	Alignment	not modelled	6.3	33	**PDB header:**apoptosis<br>**Chain:** A: **PDB Molecule:**activator of apoptosis harakiri;<br>**PDBTitle:** solution structure of the transmembrane domain of bcl-2 member2 harakiri in micelles<br>**PDB Entry:** PDBe RCSB PDBj
86	c3ktdC_	Alignment	not modelled	6.3	18	**PDB header:**oxidoreductase<br>**Chain:** C: **PDB Molecule:**prephenate dehydrogenase;<br>**PDBTitle:** crystal structure of a putative prephenate dehydrogenase (cgl0226)2 from corynebacterium glutamicum atcc 13032 at 2.60 a resolution<br>**PDB Entry:** PDBe RCSB PDBj
87	d1jb7a3	Alignment	not modelled	6.2	52	**Fold:** OB-fold<br>**Superfamily:** Nucleic acid-binding proteins<br>**Family:** Single strand DNA-binding domain, SSB<br>**PDB entry:** PDBe RCSB PDBj
88	c3f0iA_	Alignment	not modelled	6.2	26	**PDB header:**oxidoreductase<br>**Chain:** A: **PDB Molecule:**arsenate reductase;<br>**PDBTitle:** arsenate reductase from vibrio cholerae.<br>**PDB Entry:** PDBe RCSB PDBj
89	c3m6mF_	Alignment	not modelled	6.2	24	**PDB header:**lyase/transferase<br>**Chain:** F: **PDB Molecule:**sensory/regulatory protein rpfc;<br>**PDBTitle:** crystal structure of rpff complexed with rec domain of rpfc<br>**PDB Entry:** PDBe RCSB PDBj
90	d1pjwa_	Alignment	not modelled	6.0	42	**Fold:** Immunoglobulin-like beta-sandwich<br>**Superfamily:** E set domains<br>**Family:** Class II viral fusion proteins C-terminal domain<br>**PDB entry:** PDBe RCSB PDBj
91	c6nryA_	Alignment	not modelled	6.0	18	**PDB header:**immune system<br>**Chain:** A: **PDB Molecule:**caspase-4;<br>**PDBTitle:** crystal structure of human caspase-4<br>**PDB Entry:** PDBe RCSB PDBj
92	c4kr3A_	Alignment	not modelled	5.8	31	**PDB header:**ligase/rna<br>**Chain:** A: **PDB Molecule:**glycine--trna ligase;<br>**PDBTitle:** glycyl-trna synthetase mutant e71g in complex with trna-gly<br>**PDB Entry:** PDBe RCSB PDBj
93	c3p45I_	Alignment	not modelled	5.8	24	**PDB header:**hydrolase<br>**Chain:** I: **PDB Molecule:**caspase-6;<br>**PDBTitle:** crystal structure of apo-caspase-6 at physiological ph<br>**PDB Entry:** PDBe RCSB PDBj
94	c3e35A_	Alignment	not modelled	5.8	26	**PDB header:**unknown function<br>**Chain:** A: **PDB Molecule:**uncharacterized protein sco1997;<br>**PDBTitle:** actinobacteria-specific protein of unknown function, sco1997<br>**PDB Entry:** PDBe RCSB PDBj
95	c2wl8D_	Alignment	not modelled	5.8	47	**PDB header:**protein transport<br>**Chain:** D: **PDB Molecule:**peroxisomal biogenesis factor 19;

95	[illegible]	Alignment	not modelled	5.8	47	**PDBTitle:** x-ray crystal structure of pex19p<br>**PDB Entry:** PDBe RCSB PDBj
96	c2pheC_	Alignment	not modelled	5.8	40	**PDB header:**transcription<br>**Chain:** C: **PDB Molecule:**alpha trans-inducing protein;<br>**PDBTitle:** model for vp16 binding to pc4<br>**PDB Entry:** PDBe RCSB PDBj
97	c6pdqA_	Alignment	not modelled	5.8	25	**PDB header:**apoptosis<br>**Chain:** A: **PDB Molecule:**ancestral effector caspase-3/6/7;<br>**PDBTitle:** ancestral effector caspase 3/6/7<br>**PDB Entry:** PDBe RCSB PDBj
98	c3edqC_	Alignment	not modelled	5.7	21	**PDB header:**hydrolase/hydrolase inhibitor<br>**Chain:** C: **PDB Molecule:**caspase-3;<br>**PDBTitle:** crystal structure of caspase-3 with inhibitor ac-ldesd-cho<br>**PDB Entry:** PDBe RCSB PDBj
99	c1i51A_	Alignment	not modelled	5.7	26	**PDB header:**hydrolase/hydrolase inhibitor<br>**Chain:** A: **PDB Molecule:**caspase-7 subunit p20;<br>**PDBTitle:** crystal structure of caspase-7 complexed with xiap<br>**PDB Entry:** PDBe RCSB PDBj
