# Supplementary material for: Novel RNA viruses associated with avian haemosporidian parasites
Source: PLoS One. 2022 Jun 30;17(6):e0269881. doi: 10.1371/journal.pone.0269881 (PMC9246168; doi:10.1371/journal.pone.0269881)
Supplement: S1 Appendix — Results from diamond BLASTx using two databases provided, and an E-value cutoff of 1E-10. Includes Trinity assembly stats report for all transcriptomes used in this study. IQ-Tree files include Newick format tree file and aligned sequences used for analysis. (ZIP) [file pone.0269881.s004.zip › S1Appendix/Phyre2_pdb_files/MaRNAV3_info_tables/ss_report_MaRNAV3.pdf]

Phyre2

Email	jrodri17@mail.sfsu.edu
Description	MaRNAV3__
Date	Wed Feb 2 22:32:59 GMT 2022
Unique Job ID	3d381ef96b5bd38c

Secondary structure and disorder prediction

	1–60
Sequence	M E E Y R V I Q A T H L R D V V G N K V S C V F Y H L L P T L S R P K A R R N D N L W L Y Y I P E G S L L S M K V K N K
Disorder	? ? ? ? ? ? ? — ? ? ? ? ? — ? — ? ? ? ?

	61–120
Sequence	T V R S Q K W E K G D T F S H R Q S R A L Y V V L T R N L G L T K A S A K I L I N R G K L H L S R I E G V I S G I A D A
Disorder	? ? ? ? ? ? ? ? ? ? ? ? ? — ? — ? — ? ? ? — ? — ? —

	121–180
Sequence	M L C A D P F I F N I E N V E N W G I L K D L C R K V F Q V G I T N L D L L V D S W K E W T R W L F H Q H A K T D V I G
Disorder	—

	181–240
Sequence	E L V K P A K N N I Y V R L N K L S Q I Q E L K D D F F S F Q K L S H I I S T R Q F P Y M G K R R E E K S L K R F E E V
Disorder	— ? — ? ? ? ? —

	241–300
Sequence	V T S I H N V S D L H H C E M A S A A F Y I G R Y C R R Y P R Y R N A S H F S I N S S G D F H F T I G E G G N A V A T R
Disorder	— ? — ? —

	301–360
Sequence	T L A I Q V L N R R P T E E V E D T P W G I L H H N Q N M E I W R Y L F R P E P I T E G E F L K P M V S G F P K E Q P H
Disorder	— ? —

	361–420
Sequence	R Y Y G L D E H F G K Q L L Y A A W K V K E S I P K V R V S T V P E M G N K A R I V T I G P A W L Q I I Q A P L A H Q L
Disorder	— ? — ? — ? ? — ? ? ? ? ? ? ? ? ? ? — ? ? —

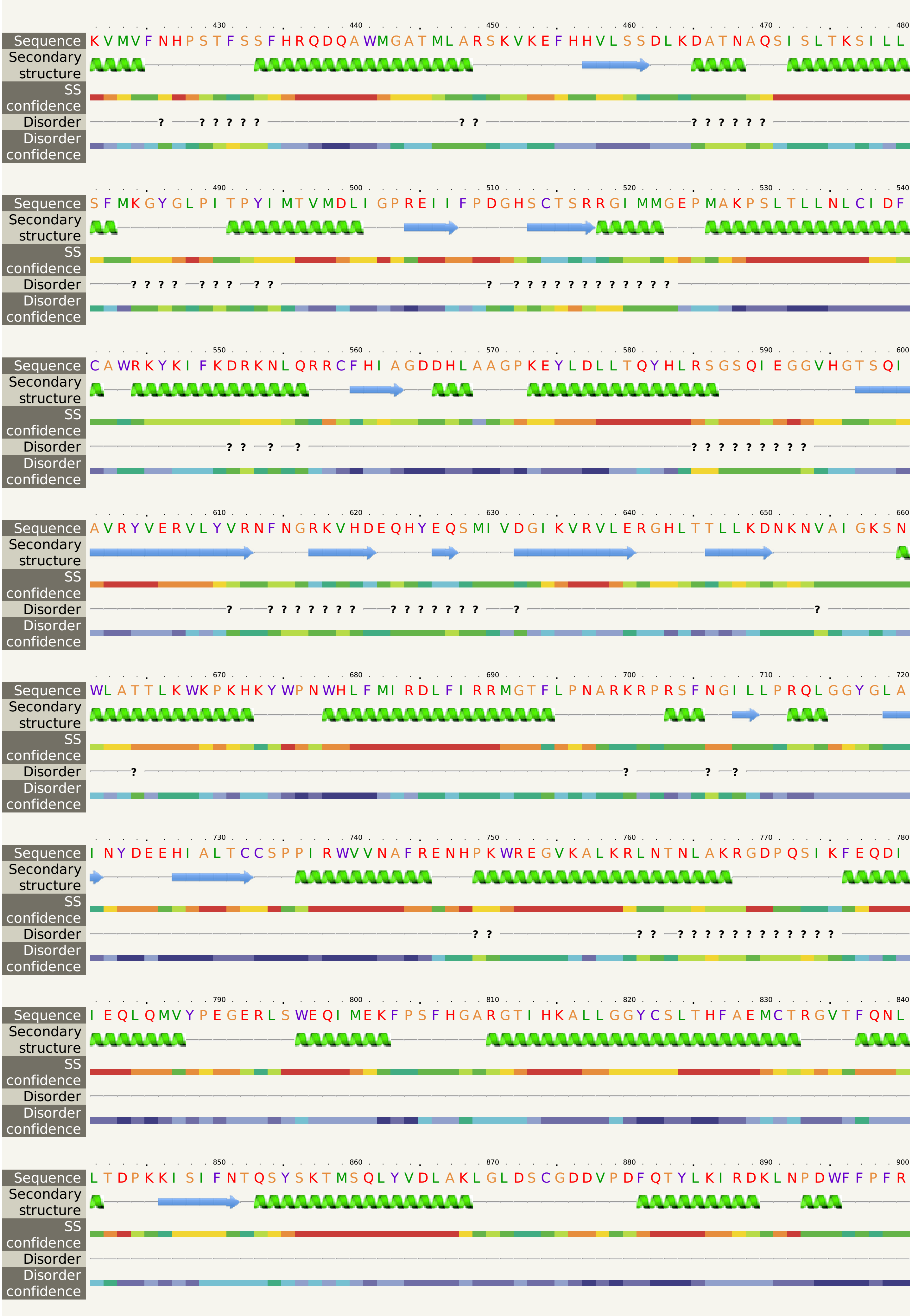

430 440 450 460 470 480
Sequence KVMVFNHPSTFSSFHRQDQAWMGATMLARSKVKEFHHVLSSDLKDATNAQSISLTKSILL
Secondary structure
SS confidence
Disorder
Disorder confidence
490 500 510 520 530 540
Sequence SFMKGYGLPITPYIMTVMDLIGPREIIFPDGHSCTSRRGIMMGEPMAKPSLTLLNLCIDF
Secondary structure
SS confidence
Disorder
Disorder confidence
550 560 570 580 590 600
Sequence CAWRKYKIFKDRKNLQRRCFHIAGDDHLAAGPKEYLDLLTQYHLRSGSQIEGGVHGTSQI
Secondary structure
SS confidence
Disorder
Disorder confidence
610 620 630 640 650 660
Sequence AVRYVERVLYVRNFNGRKVHDEQHYEQSMIVDGIKVRVLERGHLTTLLKDNKNVAIGKSN
Secondary structure
SS confidence
Disorder
Disorder confidence
670 680 690 700 710 720
Sequence WLATTLKWKPKHKYWPNWHLFMIRDLFIRRMGTFLPNARKRPRSFNGILLPRQLGGYGLA
Secondary structure
SS confidence
Disorder
Disorder confidence
730 740 750 760 770 780
Sequence INYDEEHIALTCCSPPIRWVVNAFRENHPKWREGVKALKRLNTNLAKRGDPQSIKFEQDI
Secondary structure
SS confidence
Disorder
Disorder confidence
790 800 810 820 830 840
Sequence IEQLQMVYPEGERLSWEQIMEKFPSFHGARGTIHKALLGGYCSLTHFAEMCTRGVTFQNL
Secondary structure
SS confidence
Disorder
Disorder confidence
850 860 870 880 890 900
Sequence LTDPKKISIFNTQSYSKTMSQLYVDLAKLGLDSCGDDVPDFQTYLKIRDKLNPDWFFPFR
Secondary structure
SS confidence
Disorder
Disorder confidence

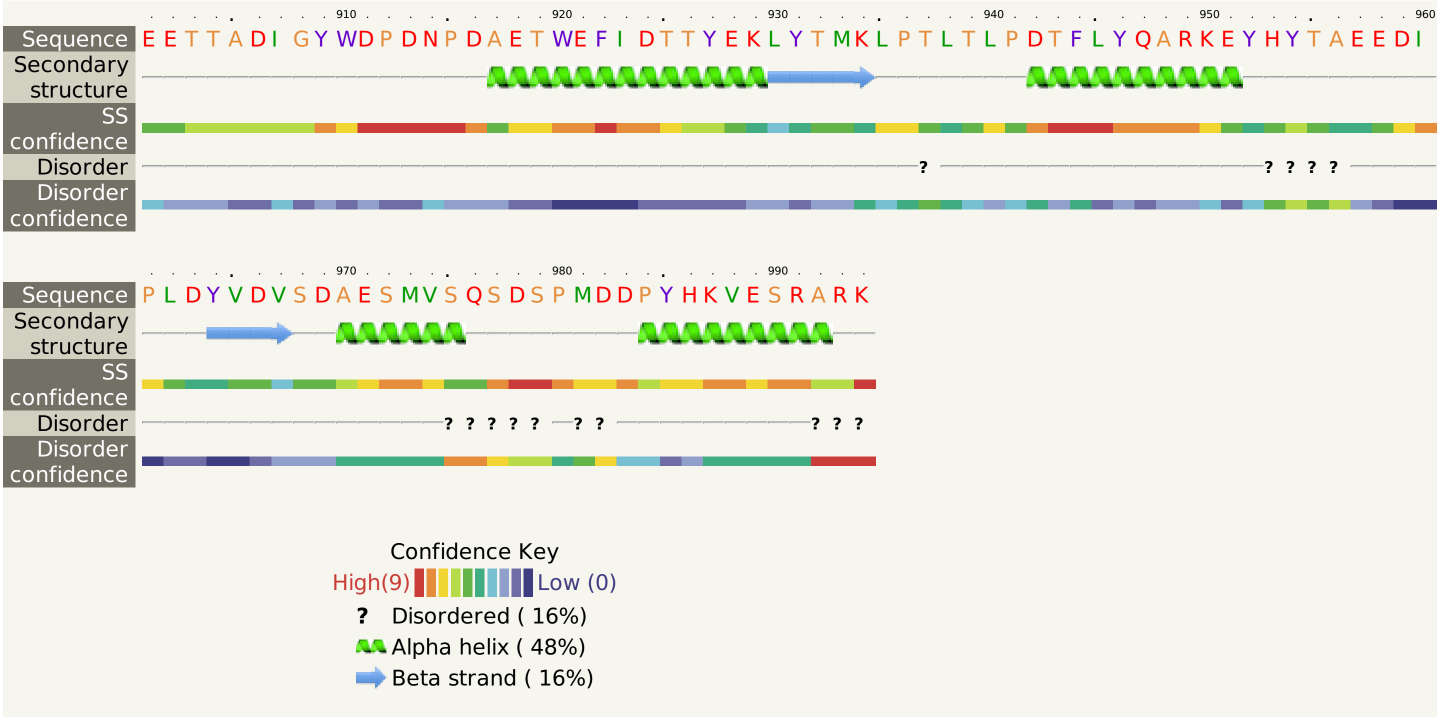

910
920
930
940
950
960
Sequence
EETTADIGYWDPDNPDAETWEFIDTTYEKLYTMKLPTLTLPDTFLYQARKEYHYTAEEDI
Secondary structure
SS confidence
Disorder
Disorder confidence
970
980
990
Sequence
PLDYVDVSDAESMVSQSDSPMDDPYHKVESRARK
Secondary structure
SS confidence
Disorder
Disorder confidence
Confidence Key
High(9)
Low (0)
? Disordered (16%)
Alpha helix (48%)
Beta strand (16%)
