# Supplementary material for: Novel RNA viruses associated with avian haemosporidian parasites
Source: PLoS One. 2022 Jun 30;17(6):e0269881. doi: 10.1371/journal.pone.0269881 (PMC9246168; doi:10.1371/journal.pone.0269881)
Supplement: S1 Appendix — Results from diamond BLASTx using two databases provided, and an E-value cutoff of 1E-10. Includes Trinity assembly stats report for all transcriptomes used in this study. IQ-Tree files include Newick format tree file and aligned sequences used for analysis. (ZIP) [file pone.0269881.s004.zip › S1Appendix/Phyre2_pdb_files/MaRNAV3_info_tables/hit_report_MaRNAV3.pdf]

Phyre2

Email	jrodri17@mail.sfsu.edu
Description	MaRNAV3__
Date	Wed Feb 2 22:32:59 GMT 2022
Unique Job ID	3d381ef96b5bd38c

Detailed template information

#	Template	Alignment Coverage	3D Model	Confidence	% i.d.	Template Information
1	d1u09a_	Alignment	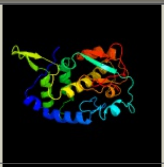	95.6	15	**Fold:** DNA/RNA polymerases<br>**Superfamily:** DNA/RNA polymerases<br>**Family:** RNA-dependent RNA-polymerase<br>**PDB entry:** PDBe RCSB PDBj
2	d1khva_	Alignment	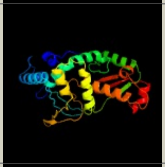	95.3	13	**Fold:** DNA/RNA polymerases<br>**Superfamily:** DNA/RNA polymerases<br>**Family:** RNA-dependent RNA-polymerase<br>**PDB entry:** PDBe RCSB PDBj
3	c6r1iB_	Alignment	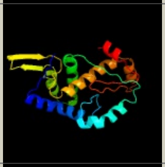	94.5	15	**PDB header:**transferase<br>**Chain:** B: **PDB Molecule:**genome polyprotein;<br>**PDBTitle:** structure of porcine aichi virus polymerase<br>**PDB Entry:** PDBe RCSB PDBj
4	d1xr6a_	Alignment	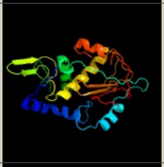	94.4	12	**Fold:** DNA/RNA polymerases<br>**Superfamily:** DNA/RNA polymerases<br>**Family:** RNA-dependent RNA-polymerase<br>**PDB entry:** PDBe RCSB PDBj
5	c3n6mA_	Alignment	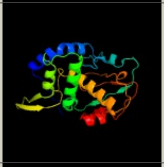	94.3	13	**PDB header:**transferase<br>**Chain:** A: **PDB Molecule:**rna-dependent rna polymerase;<br>**PDBTitle:** crystal structure of ev71 rdrp in complex with gtp<br>**PDB Entry:** PDBe RCSB PDBj
6	c3nahC_	Alignment	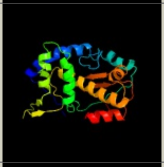	94.1	16	**PDB header:**transferase<br>**Chain:** C: **PDB Molecule:**rna dependent rna polymerase;<br>**PDBTitle:** crystal structures and functional analysis of murine norovirus rna-2 dependent rna polymerase<br>**PDB Entry:** PDBe RCSB PDBj
7	c3mmpG_	Alignment	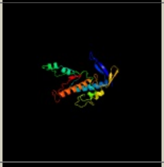	93.2	22	**PDB header:**transferase<br>**Chain:** G: **PDB Molecule:**rna-directed rna polymerase beta chain;<br>**PDBTitle:** structure of the qb replicase, an rna-dependent rna polymerase2 consisting of viral and host proteins<br>**PDB Entry:** PDBe RCSB PDBj
8	c5y6rA_	Alignment	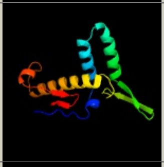	93.0	16	**PDB header:**transferase<br>**Chain:** A: **PDB Molecule:**genome polyprotein;<br>**PDBTitle:** crystal structure of csfv ns5b<br>**PDB Entry:** PDBe RCSB PDBj
9	c4nz0F_	Alignment	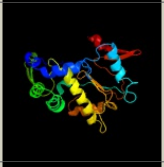	92.5	16	**PDB header:**transferase<br>**Chain:** F: **PDB Molecule:**genome polyprotein;<br>**PDBTitle:** the emcv 3dpol structure at 2.8a resolution<br>**PDB Entry:** PDBe RCSB PDBj
10	d1xr7a_	Alignment	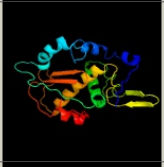	91.7	12	**Fold:** DNA/RNA polymerases<br>**Superfamily:** DNA/RNA polymerases<br>**Family:** RNA-dependent RNA-polymerase<br>**PDB entry:** PDBe RCSB PDBj
11	d1s48a_	Alignment	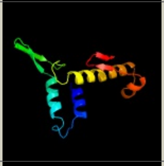	91.0	19	**Fold:** DNA/RNA polymerases<br>**Superfamily:** DNA/RNA polymerases<br>**Family:** RNA-dependent RNA-polymerase<br>**PDB entry:** PDBe RCSB PDBj

12	c2b43D_	Alignment	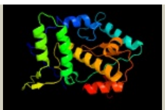	88.8	14	**PDB header:**viral protein<br>**Chain:** D: **PDB Molecule:**non-structural polyprotein;<br>**PDBTitle:** crystal structure of the norwalk virus rna dependent rna polymerase2 from strain hu/nlv/dresden174/1997/ge<br>**PDB Entry:** PDBe RCSB PDBj
13	c2uutA_	Alignment	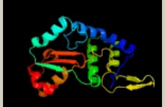	88.2	10	**PDB header:**hydrolase<br>**Chain:** A: **PDB Molecule:**rna-directed rna polymerase;<br>**PDBTitle:** the 2.4 angstrom resolution structure of the d346g mutant of the2 sapporo virus rdrp polymerase<br>**PDB Entry:** PDBe RCSB PDBj
14	d1sh0a_	Alignment	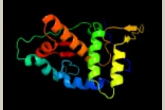	86.6	13	**Fold:** DNA/RNA polymerases<br>**Superfamily:** DNA/RNA polymerases<br>**Family:** RNA-dependent RNA-polymerase<br>**PDB entry:** PDBe RCSB PDBj
15	c2ijd1_	Alignment	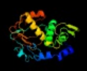	86.4	15	**PDB header:**hydrolase, transferase<br>**Chain:** 1: **PDB Molecule:**picornain 3c, rna-directed rna polymerase;<br>**PDBTitle:** crystal structure of the poliovirus precursor protein 3cd<br>**PDB Entry:** PDBe RCSB PDBj
16	d1ra6a_	Alignment	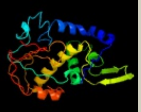	80.0	16	**Fold:** DNA/RNA polymerases<br>**Superfamily:** DNA/RNA polymerases<br>**Family:** RNA-dependent RNA-polymerase<br>**PDB entry:** PDBe RCSB PDBj
17	c6qwtA_	Alignment	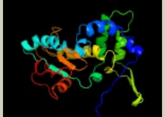	76.3	16	**PDB header:**viral protein<br>**Chain:** A: **PDB Molecule:**genome polyprotein;<br>**PDBTitle:** sicinivirus 3dpol rna dependent rna polymerase<br>**PDB Entry:** PDBe RCSB PDBj
18	d1xr5a_	Alignment	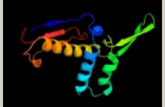	72.1	15	**Fold:** DNA/RNA polymerases<br>**Superfamily:** DNA/RNA polymerases<br>**Family:** RNA-dependent RNA-polymerase<br>**PDB entry:** PDBe RCSB PDBj
19	c3aggA_	Alignment	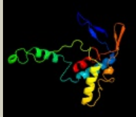	60.0	24	**PDB header:**translation,transferase<br>**Chain:** A: **PDB Molecule:**elongation factor ts, elongation factor tu 1, linker, q<br>**PDBTitle:** structure of viral polymerase form ii<br>**PDB Entry:** PDBe RCSB PDBj
20	c3wzsA_	Alignment	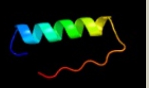	59.2	34	**PDB header:**transferase<br>**Chain:** A: **PDB Molecule:**udp-glucose-glycoprotein glucosyltransferase-like protein;<br>**PDBTitle:** crystal structure of trx3 domain of uggt (detergent-bound form)<br>**PDB Entry:** PDBe RCSB PDBj
21	c5i62A_	Alignment	not modelled	49.4	16	**PDB header:**viral protein, replication<br>**Chain:** A: **PDB Molecule:**potential rna-dependent rna polymerase;<br>**PDBTitle:** crystal structure of the insertion loop deletion mutant of the rna-2 dependent rna polymerase of a human picorbirnavirus<br>**PDB Entry:** PDBe RCSB PDBj
22	c4v194_	Alignment	not modelled	46.4	27	**PDB header:**ribosome<br>**Chain:** 4: **PDB Molecule:**mitoribosomal protein bl31m, mrpl55;<br>**PDBTitle:** structure of the large subunit of the mammalian mitoribosome, part 12 of 2<br>**PDB Entry:** PDBe RCSB PDBj
23	d1nb4a_	Alignment	not modelled	41.9	23	**Fold:** DNA/RNA polymerases<br>**Superfamily:** DNA/RNA polymerases<br>**Family:** RNA-dependent RNA-polymerase<br>**PDB entry:** PDBe RCSB PDBj
24	d1tp6a_	Alignment	not modelled	39.8	40	**Fold:** Cystatin-like<br>**Superfamily:** NTF2-like<br>**Family:** PA1314-like<br>**PDB entry:** PDBe RCSB PDBj
25	c3riuC_	Alignment	not modelled	38.3	17	**PDB header:**hydrolase<br>**Chain:** C: **PDB Molecule:**translin associated factor x, isoform b;<br>**PDBTitle:** crystal structure of drosophila hexameric c3po formed by truncated2 translin and trax<br>**PDB Entry:** PDBe RCSB PDBj
26	d1gx5a_	Alignment	not modelled	35.4	27	**Fold:** DNA/RNA polymerases<br>**Superfamily:** DNA/RNA polymerases<br>**Family:** RNA-dependent RNA-polymerase<br>**PDB entry:** PDBe RCSB PDBj
27	c2cjqA_	Alignment	not modelled	33.7	26	**PDB header:**transferase<br>**Chain:** A: **PDB Molecule:**rna-directed rna polymerase;<br>**PDBTitle:** bovine viral diarrhea virus cp7-r12 rna-dependent rna2 polymerase<br>**PDB Entry:** PDBe RCSB PDBj
						**PDB header:**protein binding/protein transport<br>**Chain:** B: **PDB Molecule:**pals1-associated tight junction protein;

28	c1vf6B_	Alignment	not modelled	29.1	36	**PDBTitle:** 2.1 angstrom crystal structure of the pals-1-l27n and patj l272 heterodimer complex<br>**PDB Entry:** PDBe RCSB PDBj
29	d1vf6a_	Alignment	not modelled	28.3	36	**Fold:** L27 domain<br>**Superfamily:** L27 domain<br>**Family:** L27 domain<br>**PDB entry:** PDBe RCSB PDBj
30	d1raja_	Alignment	not modelled	28.2	20	**Fold:** DNA/RNA polymerases<br>**Superfamily:** DNA/RNA polymerases<br>**Family:** RNA-dependent RNA-polymerase<br>**PDB entry:** PDBe RCSB PDBj
31	c2qrxA_	Alignment	not modelled	23.6	20	**PDB header:**dna binding protein<br>**Chain:** A: **PDB Molecule:**gm27569p;<br>**PDBTitle:** crystal structure of drosophila melanogaster translin protein<br>**PDB Entry:** PDBe RCSB PDBj
32	c5kgqA_	Alignment	not modelled	23.2	50	**PDB header:**unknown function<br>**Chain:** A: **PDB Molecule:**uncharacterized protein;<br>**PDBTitle:** nmr structure and dynamics of q4dy78, a conserved kinetoplasid-2 specific protein from trypanosoma cruzi<br>**PDB Entry:** PDBe RCSB PDBj
33	c3axjB_	Alignment	not modelled	22.9	38	**PDB header:**dna binding protein<br>**Chain:** B: **PDB Molecule:**translin associated factor x, isoform b;<br>**PDBTitle:** high resolution crystal structure of c3po<br>**PDB Entry:** PDBe RCSB PDBj
34	c3zc1E_	Alignment	not modelled	21.8	13	**PDB header:**hydrolase<br>**Chain:** E: **PDB Molecule:**aftrax;<br>**PDBTitle:** crystal structure of afc3po<br>**PDB Entry:** PDBe RCSB PDBj
35	c3f8mA_	Alignment	not modelled	21.4	24	**PDB header:**transcription<br>**Chain:** A: **PDB Molecule:**gntr-family protein transcriptional regulator;<br>**PDBTitle:** crystal structure of phnf from mycobacterium smegmatis<br>**PDB Entry:** PDBe RCSB PDBj
36	c5hhlA_	Alignment	not modelled	21.0	14	**PDB header:**rna binding protein<br>**Chain:** A: **PDB Molecule:**retron-type reverse transcriptase;<br>**PDBTitle:** reverse transcriptase domain of group ii intron maturase from2 eubacterium rectale in p21 space group<br>**PDB Entry:** PDBe RCSB PDBj
37	d2ha9a1	Alignment	not modelled	20.2	19	**Fold:** PFL-like glycyl radical enzymes<br>**Superfamily:** PFL-like glycyl radical enzymes<br>**Family:** SP0239-like<br>**PDB entry:** PDBe RCSB PDBj
38	c7eepO_	Alignment	not modelled	19.9	28	**PDB header:**virus<br>**Chain:** O: **PDB Molecule:**pam1 adaptor proteins;<br>**PDBTitle:** cyanophage pam1 portal-adaptor complex<br>**PDB Entry:** PDBe RCSB PDBj
39	c3s6pC_	Alignment	not modelled	19.6	30	**PDB header:**virus<br>**Chain:** C: **PDB Molecule:**capsid protein;<br>**PDBTitle:** crystal structure of helicoverpa armigera stunt virus<br>**PDB Entry:** PDBe RCSB PDBj
40	c3gwlB_	Alignment	not modelled	19.1	19	**PDB header:**oxidoreductase<br>**Chain:** B: **PDB Molecule:**fad-linked sulfhydryl oxidase;<br>**PDBTitle:** crystal structure of asfv pb119l, a viral sulfhydryl oxidase<br>**PDB Entry:** PDBe RCSB PDBj
41	c6z0fB_	Alignment	not modelled	18.8	18	**PDB header:**membrane protein<br>**Chain:** B: **PDB Molecule:**esx secretion system protein yukc;<br>**PDBTitle:** crystal structure of the membrane pseudokinase yukc/essb from bacillus2 subtilis t7ss<br>**PDB Entry:** PDBe RCSB PDBj
42	d1y76a1	Alignment	not modelled	18.8	36	**Fold:** L27 domain<br>**Superfamily:** L27 domain<br>**Family:** L27 domain<br>**PDB entry:** PDBe RCSB PDBj
43	d2pyqa1	Alignment	not modelled	18.5	31	**Fold:** Jann4075-like<br>**Superfamily:** Jann4075-like<br>**Family:** Jann4075-like<br>**PDB entry:** PDBe RCSB PDBj
44	c2dvlB_	Alignment	not modelled	18.3	20	**PDB header:**oxidoreductase<br>**Chain:** B: **PDB Molecule:**acyl-coa dehydrogenase;<br>**PDBTitle:** crystal structure of project tt0160 from thermus thermophilus hb8<br>**PDB Entry:** PDBe RCSB PDBj
45	c6zfbd_	Alignment	not modelled	18.3	28	**PDB header:**transcription<br>**Chain:** D: **PDB Molecule:**dna-directed rna polymerase subunit delta;<br>**PDBTitle:** structure of the b. subtilis rna polymerase in complex with held2 (dimer)<br>**PDB Entry:** PDBe RCSB PDBj
46	c6zfbD_	Alignment	not modelled	18.3	28	**PDB header:**transcription<br>**Chain:** D: **PDB Molecule:**dna-directed rna polymerase subunit delta;<br>**PDBTitle:** structure of the b. subtilis rna polymerase in complex with held2 (dimer)<br>**PDB Entry:** PDBe RCSB PDBj
47	c6s9uA_	Alignment	not modelled	18.1	21	**PDB header:**transferase<br>**Chain:** A: **PDB Molecule:**putative sucrose phosphorylase;<br>**PDBTitle:** crystal structure of sucrose 6f-phosphate phosphorylase from2 ilumatobacter coccineus<br>**PDB Entry:** PDBe RCSB PDBj
48	d1qvxa_	Alignment	not modelled	17.8	16	**Fold:** Four-helical up-and-down bundle<br>**Superfamily:** FAT domain of focal adhesion kinase<br>**Family:** FAT domain of focal adhesion kinase<br>**PDB entry:** PDBe RCSB PDBj
49	c2krcA_	Alignment	not modelled	17.8	26	**PDB header:**transcription<br>**Chain:** A: **PDB Molecule:**dna-directed rna polymerase subunit delta;<br>**PDBTitle:** solution structure of the n-terminal domain of bacillus2 subtilis delta subunit of rna polymerase<br>**PDB Entry:** PDBe RCSB PDBj
50	c1f0cB_	Alignment	not modelled	17.7	57	**PDB header:**viral protein<br>**Chain:** B: **PDB Molecule:**ice inhibitor;<br>**PDBTitle:** structure of the viral serpin crma

						**PDB Entry:** PDBe RCSB PDBj
51	c1e2xA_	Alignment	not modelled	17.2	16	**PDB header:**transcriptional regulation<br>**Chain:** A: **PDB Molecule:**fatty acid metabolism regulator protein;<br>**PDBTitle:** fadr, fatty acid responsive transcription factor from e.2 coli<br>**PDB Entry:** PDBe RCSB PDBj
52	c6l7qA_	Alignment	not modelled	17.2	30	**PDB header:**unknown function<br>**Chain:** A: **PDB Molecule:**hypothetical protein;<br>**PDBTitle:** crystal structure of a hypothetical protein pych_01220 derived from2 pyrococcus yayanosii<br>**PDB Entry:** PDBe RCSB PDBj
53	c3pjaK_	Alignment	not modelled	17.1	33	**PDB header:**hydrolase<br>**Chain:** K: **PDB Molecule:**translin-associated protein x;<br>**PDBTitle:** crystal structure of human c3po complex<br>**PDB Entry:** PDBe RCSB PDBj
54	d1k04a_	Alignment	not modelled	16.6	19	**Fold:** Four-helical up-and-down bundle<br>**Superfamily:** FAT domain of focal adhesion kinase<br>**Family:** FAT domain of focal adhesion kinase<br>**PDB entry:** PDBe RCSB PDBj
55	c3qd1X_	Alignment	not modelled	16.5	22	**PDB header:**sugar binding protein<br>**Chain:** X: **PDB Molecule:**platelet binding protein gspb;<br>**PDBTitle:** gspb plus alpha-2,3-sialyl (1-thioethyl)galactose<br>**PDB Entry:** PDBe RCSB PDBj
56	c6gcsU_	Alignment	not modelled	16.2	40	**PDB header:**oxidoreductase<br>**Chain:** U: **PDB Molecule:**nupm subunit;<br>**PDBTitle:** cryo-em structure of respiratory complex i from yarrowia lipolytica<br>**PDB Entry:** PDBe RCSB PDBj
57	d1j1ja_	Alignment	not modelled	16.1	23	**Fold:** alpha-alpha superhelix<br>**Superfamily:** Translin<br>**Family:** Translin<br>**PDB entry:** PDBe RCSB PDBj
58	c4dg7D_	Alignment	not modelled	15.6	20	**PDB header:**dna binding protein<br>**Chain:** D: **PDB Molecule:**gm27569p;<br>**PDBTitle:** low resolution structure of drosophila translin<br>**PDB Entry:** PDBe RCSB PDBj
59	d1xmta_	Alignment	not modelled	15.5	24	**Fold:** Acyl-CoA N-acyltransferases (Nat)<br>**Superfamily:** Acyl-CoA N-acyltransferases (Nat)<br>**Family:** N-acetyl transferase, NAT<br>**PDB entry:** PDBe RCSB PDBj
60	c4iu8A_	Alignment	not modelled	15.2	54	**PDB header:**transport protein<br>**Chain:** A: **PDB Molecule:**nitrite extrusion protein 2;<br>**PDBTitle:** crystal structure of a membrane transporter (selenomethionine2 derivative)<br>**PDB Entry:** PDBe RCSB PDBj
61	d1qxma1	Alignment	not modelled	14.9	20	**Fold:** beta-Trefoil<br>**Superfamily:** Ricin B-like lectins<br>**Family:** Ricin B-like<br>**PDB entry:** PDBe RCSB PDBj
62	c4xpuA_	Alignment	not modelled	14.8	22	**PDB header:**hydrolase<br>**Chain:** A: **PDB Molecule:**endonuclease v;<br>**PDBTitle:** the crystal structure of endov from e.coli<br>**PDB Entry:** PDBe RCSB PDBj
63	d1ny722	Alignment	not modelled	14.6	27	**Fold:** Nucleoplasmin-like/VP (viral coat and capsid proteins)<br>**Superfamily:** Positive stranded ssRNA viruses<br>**Family:** Comoviridae-like VP<br>**PDB entry:** PDBe RCSB PDBj
64	c2cjjA_	Alignment	not modelled	14.5	17	**PDB header:**dna binding protein<br>**Chain:** A: **PDB Molecule:**radialis;<br>**PDBTitle:** crystal structure of the myb domain of the rad transcription factor2 from antirrhinum majus<br>**PDB Entry:** PDBe RCSB PDBj
65	d2cjja1	Alignment	not modelled	14.5	17	**Fold:** DNA/RNA-binding 3-helical bundle<br>**Superfamily:** Homeodomain-like<br>**Family:** Myb/SANT domain<br>**PDB entry:** PDBe RCSB PDBj
66	d2v4ja2	Alignment	not modelled	14.4	25	**Fold:** Ferredoxin-like<br>**Superfamily:** Nitrite/Sulfite reductase N-terminal domain-like<br>**Family:** DsrA/DsrB N-terminal-domain-like<br>**PDB entry:** PDBe RCSB PDBj
67	d1pgl22	Alignment	not modelled	13.9	38	**Fold:** Nucleoplasmin-like/VP (viral coat and capsid proteins)<br>**Superfamily:** Positive stranded ssRNA viruses<br>**Family:** Comoviridae-like VP<br>**PDB entry:** PDBe RCSB PDBj
68	c5f5uH_	Alignment	not modelled	13.9	75	**PDB header:**splicing<br>**Chain:** H: **PDB Molecule:**putative uncharacterized protein;<br>**PDBTitle:** crystal structure of the snu23-prp38-mfap1(217-258) complex of2 chaetomium thermophilum<br>**PDB Entry:** PDBe RCSB PDBj
69	c5f5uB_	Alignment	not modelled	13.8	75	**PDB header:**splicing<br>**Chain:** B: **PDB Molecule:**putative uncharacterized protein;<br>**PDBTitle:** crystal structure of the snu23-prp38-mfap1(217-258) complex of2 chaetomium thermophilum<br>**PDB Entry:** PDBe RCSB PDBj
70	c2i1jA_	Alignment	not modelled	13.8	13	**PDB header:**cell adhesion, membrane protein<br>**Chain:** A: **PDB Molecule:**moesin;<br>**PDBTitle:** moesin from spodoptera frugiperda at 2.1 angstroms resolution<br>**PDB Entry:** PDBe RCSB PDBj
71	c5jreC_	Alignment	not modelled	13.7	25	**PDB header:**dna binding protein<br>**Chain:** C: **PDB Molecule:**neq131;<br>**PDBTitle:** crystal structure of nec3po in complex with ssdna.<br>**PDB Entry:** PDBe RCSB PDBj
72	c5jjpB_	Alignment	not modelled	13.7	37	**PDB header:**ligase<br>**Chain:** B: **PDB Molecule:**nonribosomal peptide synthase;<br>**PDBTitle:** crystal structure of cmis6<br>**PDB Entry:** PDBe RCSB PDBj
73	d3bu7a1	Alignment	not modelled	13.7	20	**Fold:** Double-stranded beta-helix<br>**Superfamily:** RmlC-like cupins<br>**Family:** Gentisate 1,2-dioxygenase-like<br>**PDB entry:** PDBe RCSB PDBj
						**PDB header:**oxidoreductase<br>**Chain:** A: **PDB Molecule:**gentisate 1,2-dioxygenase;

74	c3bu7A_	Alignment	not modelled	13.7	20	**PDBTitle:** crystal structure and biochemical characterization of gdosp, a2 gentisate 1,2-dioxygenase from silicibacter pomeroyi<br>**PDB Entry:** PDBe RCSB PDBj
75	c2l5bA_	Alignment	not modelled	13.6	25	**PDB header:**apoptosis<br>**Chain:** A: **PDB Molecule:**activator of apoptosis harakiri;<br>**PDBTitle:** solution structure of the transmembrane domain of bcl-2 member2 harakiri in micelles<br>**PDB Entry:** PDBe RCSB PDBj
76	d2bo9b1	Alignment	not modelled	13.3	36	**Fold:** Cystatin-like<br>**Superfamily:** Cystatin/monellin<br>**Family:** Latexin-like<br>**PDB entry:** PDBe RCSB PDBj
77	c3gm1A_	Alignment	not modelled	13.3	16	**PDB header:**transferase<br>**Chain:** A: **PDB Molecule:**protein tyrosine kinase 2 beta;<br>**PDBTitle:** crystal structure of the focal adhesion targeting (fat) domain of pyk22 in complex with paxillin ld4 motif-derived peptides<br>**PDB Entry:** PDBe RCSB PDBj
78	c5f5tD_	Alignment	not modelled	13.1	75	**PDB header:**splicing<br>**Chain:** D: **PDB Molecule:**putative uncharacterized protein;<br>**PDBTitle:** crystal structure of the prp38-mfap1 complex of chaetomium2 thermophilum<br>**PDB Entry:** PDBe RCSB PDBj
79	c2l6gA_	Alignment	not modelled	13.0	16	**PDB header:**transferase,cell adhesion<br>**Chain:** A: **PDB Molecule:**focal adhesion kinase 1, linker, paxillin;<br>**PDBTitle:** fat-ld2 double labeled construct with free ld4 peptide<br>**PDB Entry:** PDBe RCSB PDBj
80	c6zcaD_	Alignment	not modelled	12.8	26	**PDB header:**transcription<br>**Chain:** D: **PDB Molecule:**probable dna-directed rna polymerase subunit delta,probable<br>**PDBTitle:** structure of the b. subtilis rna polymerase in complex with held2 (monomer)<br>**PDB Entry:** PDBe RCSB PDBj
81	c6s5wA_	Alignment	not modelled	12.8	16	**PDB header:**structural protein<br>**Chain:** A: **PDB Molecule:**surface protein;<br>**PDBTitle:** structure of rib domain 'rib long' from lactobacillus acidophilus<br>**PDB Entry:** PDBe RCSB PDBj
82	c2jbtA_	Alignment	not modelled	12.8	20	**PDB header:**oxidoreductase<br>**Chain:** A: **PDB Molecule:**p-hydroxyphenylacetate hydroxylase c2\:oxygenase<br>**PDBTitle:** structure of the monooxygenase component of p-2 hydroxyphenylacetate hydroxylase from acinetobacter3 baumannii<br>**PDB Entry:** PDBe RCSB PDBj
83	c6dd5B_	Alignment	not modelled	12.7	12	**PDB header:**hydrolase<br>**Chain:** B: **PDB Molecule:**mmb-1 cas6 fused to maltose binding protein,crispr-<br>**PDBTitle:** crystal structure of the cas6 domain of marinomonas mediterranea mmb-12 cas6-rt-cas1 fusion protein<br>**PDB Entry:** PDBe RCSB PDBj
84	c6idvB_	Alignment	not modelled	12.2	20	**PDB header:**ligase<br>**Chain:** B: **PDB Molecule:**peptide asparaginyl ligases;<br>**PDBTitle:** peptide asparaginyl ligases from viola yedoensis<br>**PDB Entry:** PDBe RCSB PDBj
85	d1xuba2	Alignment	not modelled	12.0	16	**Fold:** Diaminopimelate epimerase-like<br>**Superfamily:** Diaminopimelate epimerase-like<br>**Family:** PhzC/PhzF-like<br>**PDB entry:** PDBe RCSB PDBj
86	d2ra7a1	Alignment	not modelled	12.0	19	**Fold:** Four-helical up-and-down bundle<br>**Superfamily:** FAT domain of focal adhesion kinase<br>**Family:** FAT domain of focal adhesion kinase<br>**PDB entry:** PDBe RCSB PDBj
87	c2l6hA_	Alignment	not modelled	11.8	16	**PDB header:**transferase,cell adhesion<br>**Chain:** A: **PDB Molecule:**focal adhesion kinase 1, linker, paxillin;<br>**PDBTitle:** fat domain of focal adhesion kinase tethered to ld4 motif of paxillin2 via ggs linker<br>**PDB Entry:** PDBe RCSB PDBj
88	c6jleE_	Alignment	not modelled	11.7	38	**PDB header:**motor protein<br>**Chain:** E: **PDB Molecule:**myosin-iiia;<br>**PDBTitle:** crystal structure of morn4/myo3a complex<br>**PDB Entry:** PDBe RCSB PDBj
89	c4rm9A_	Alignment	not modelled	11.6	14	**PDB header:**peptide binding protein<br>**Chain:** A: **PDB Molecule:**ezrin;<br>**PDBTitle:** crystal structure of human ezrin in space group c2221<br>**PDB Entry:** PDBe RCSB PDBj
90	c3mkhC_	Alignment	not modelled	11.5	20	**PDB header:**oxidoreductase<br>**Chain:** C: **PDB Molecule:**nitroalkane oxidase;<br>**PDBTitle:** podospora anserina nitroalkane oxidase<br>**PDB Entry:** PDBe RCSB PDBj
91	d1ho8a_	Alignment	not modelled	11.3	15	**Fold:** alpha-alpha superhelix<br>**Superfamily:** ARM repeat<br>**Family:** Regulatory subunit H of the V-type ATPase<br>**PDB entry:** PDBe RCSB PDBj
92	c3dkbA_	Alignment	not modelled	11.0	27	**PDB header:**hydrolase<br>**Chain:** A: **PDB Molecule:**tumor necrosis factor, alpha-induced protein 3;<br>**PDBTitle:** crystal structure of a20, 2.5 angstrom<br>**PDB Entry:** PDBe RCSB PDBj
93	d1itwa_	Alignment	not modelled	10.8	25	**Fold:** Isocitrate/Isopropylmalate dehydrogenase-like<br>**Superfamily:** Isocitrate/Isopropylmalate dehydrogenase-like<br>**Family:** Monomeric isocitrate dehydrogenase<br>**PDB entry:** PDBe RCSB PDBj
94	c5f5uE_	Alignment	not modelled	10.7	75	**PDB header:**splicing<br>**Chain:** E: **PDB Molecule:**putative uncharacterized protein;<br>**PDBTitle:** crystal structure of the snu23-prp38-mfap1(217-258) complex of2 chaetomium thermophilum<br>**PDB Entry:** PDBe RCSB PDBj
95	c6wy9A_	Alignment	not modelled	10.5	33	**PDB header:**oxidoreductase<br>**Chain:** A: **PDB Molecule:**acyl-coa dehydrogenase domain protein tcur3483;<br>**PDBTitle:** tcur3481-tcur3483 steroid acad g363a variant<br>**PDB Entry:** PDBe RCSB PDBj
						**PDB header:**oxidoreductase<br>**Chain:** B: **PDB Molecule:**acyl-coa dehydrogenase;

96	c4x28B_	Alignment	not modelled	10.0	27	**PDBTitle:** crystal structure of the chse4-chse5 complex from mycobacterium2 tuberculosis<br>**PDB Entry:** PDBe RCSB PDBj
97	c5xdcB_	Alignment	not modelled	10.0	27	**PDB header:**oxidoreductase<br>**Chain:** B: **PDB Molecule:**thermophilic dibenzothiophene desulfurization enzyme c;<br>**PDBTitle:** crystal structure of indole-bound tdsc from paenibacillus sp. a11-2<br>**PDB Entry:** PDBe RCSB PDBj
98	d1u0ka2	Alignment	not modelled	9.9	11	**Fold:** Diaminopimelate epimerase-like<br>**Superfamily:** Diaminopimelate epimerase-like<br>**Family:** PhzC/PhzF-like<br>**PDB entry:** PDBe RCSB PDBj
99	c3uitA_	Alignment	not modelled	9.9	36	**PDB header:**cell adhesion<br>**Chain:** A: **PDB Molecule:**inad-like protein, maguk p55 subfamily member 5, protein<br>**PDBTitle:** overall structure of patj/pals1/mals complex<br>**PDB Entry:** PDBe RCSB PDBj
