# Supplementary material for: Novel RNA viruses associated with avian haemosporidian parasites
Source: PLoS One. 2022 Jun 30;17(6):e0269881. doi: 10.1371/journal.pone.0269881 (PMC9246168; doi:10.1371/journal.pone.0269881)
Supplement: S1 Appendix — Results from diamond BLASTx using two databases provided, and an E-value cutoff of 1E-10. Includes Trinity assembly stats report for all transcriptomes used in this study. IQ-Tree files include Newick format tree file and aligned sequences used for analysis. (ZIP) [file pone.0269881.s004.zip › S1Appendix/Phyre2_pdb_files/MaRNAV3_info_tables/dom_report_MaRNAV3.pdf]

Phyre2

Email	jrodri17@mail.sfsu.edu
Description	MaRNAV3__
Date	Wed Feb 2 22:32:59 GMT 2022
Unique Job ID	3d381ef96b5bd38c

Domain analysis

Rank	Aligned region
1	d1u09a_
2	d1khva_
3	c6r1iB_
4	d1xr6a_
5	c3n6mA_
6	c3nahC_
7	c3mmpG_
8	c5y6rA_
9	c4nz0F_
10	d1xr7a_
11	d1s48a_
12	c2b43D_
13	c2uutA_
14	d1sh0a_
15	c2ijd1_
16	d1ra6a_
17	c6qwtA_
18	d1xr5a_
19	c3aggA_
20	c3wzsA_
21	
22	
23	
24	
25	
26	
27	
28	
29	
30	
31	
32	
33	
34	
35	
36	
37	
38	
39	
40	
41	
42	
43	
44	
45	
46	
47	
48	
49	
50	
51	
52	
53	
54	
55	
56	
57	
58	

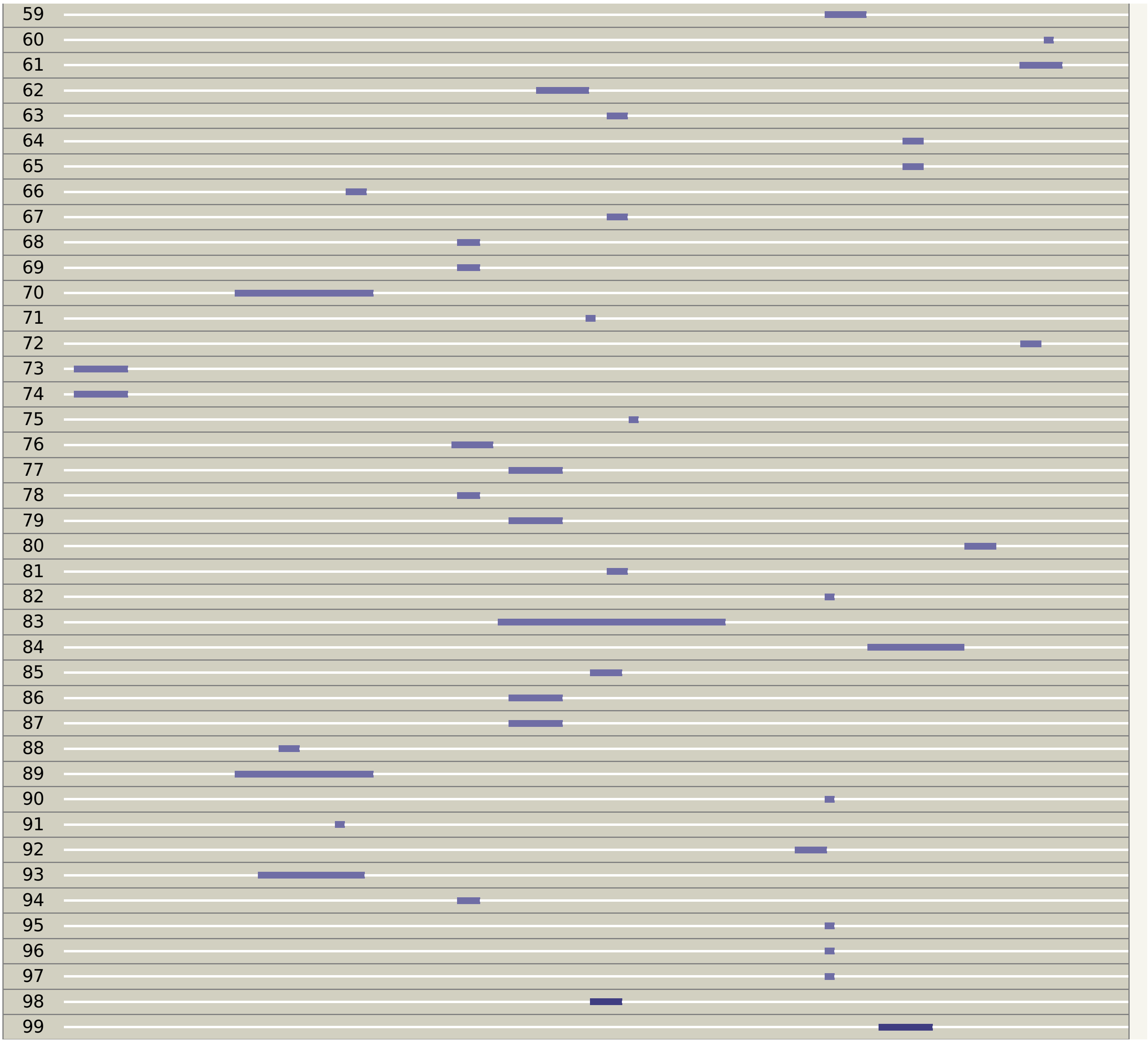
59
60
61
62
63
64
65
66
67
68
69
70
71
72
73
74
75
76
77
78
79
80
81
82
83
84
85
86
87
88
89
90
91
92
93
94
95
96
97
98
99
