# Supplementary material for: Novel RNA viruses associated with avian haemosporidian parasites
Source: PLoS One. 2022 Jun 30;17(6):e0269881. doi: 10.1371/journal.pone.0269881 (PMC9246168; doi:10.1371/journal.pone.0269881)
Supplement: Attachment — Submitted filename: Response_to_reviewers.docx [file pone.0269881.s005.docx]

Dear Dr. Pacheco,

Please consider our revised manuscript entitled “Novel RNA viruses associated with avian haemosporidian parasites” for publication in PLOS ONE.

We are grateful to you and the two anonymous reviewers for their helpful comments that have improved this manuscript. We have taken the suggestions into account and address the comments point by point below in **yellow highlight**.

We believe that this study will be of interest to a broad community of biologists studying malaria and emerging viruses.

Thank you for your consideration.

On behalf of all the authors,

Jose Roberto Rodrigues

We would like to thank reviewer #1 and #2 for their comments to improve our manuscript. Please find responses to the comments highlighted in yellow. Unless otherwise noted, the references for line numbers correspond to the clean version of the submitted paper.

**Reviewer #1:** The overall design of the study is acceptable and can conduct to the conclusion that new MaRNAV-like signals are identified as narna-like viruses and very likely infect bird hosts. Nevertheless, I would recommend to the authors to be more careful with the virus/host relationship establishment because, as well explained in the Discussion part, this study and the previous one from Charon et al are both based on bioinformatic identification. The use of "confirm" l. 197 is thus not adapted. On the other hand, some Narnaviridae are not only "suspected" fungal viruses as indicated l. 217 but have been clearly and experimentally associated to host infection (especially in S. cerevisiae).
Discussion of the identification of second segment is missing, as well as the discussion of the potential presence of the virus in the host genome. I would recommend to the authors to conduct a Blastn of their newly identified sequences to the nt NCBI to ensure the newly identified viruses are not EVEs, i.e. viral elements integrated into host genomes.

We appreciate the comments and believe we have addressed all the concerns. To address the virus/host relationship better we found other instances in the manuscript were we incorrectly used “infect” and replaced them with more accurate words. The comment on the potential presence of the virus in the host genome was addressed by performing the BLASTn search of the virus sequences on NCBI, and is delineated in the “Virus Discovery” section of the paper. We address the subsequent comments below.

Please see below for minor comments/corrections :

l.25 : Precise the taxon of parasites here ? (Apicomplexa/Haemosporidian)

The precise taxon was added.

l.29 : Does it mean MaRNAV-3 share 40% with MaRNAV-1 and MaRNAV3 share 60% with MaRNAV-2 ? Unclear...

We changed the sentence to more accurately describe the amino acid percent identities.

l.56 : I would add another ref for the protist definition and phylogeny... Perhaps Burki F. et al. (2020) ‘The New Tree of Eukaryotes’, Trends in Ecology & Evolution, 35: 43–55.

We added the citation to the paragraph.

l.76 : RdRp cannot be considered as "highly" conserved but constitutes the most conserved protein in RNA viruses.

We changed from “highly” to “most” conserved protein in RNA viruses for a more accurate description.

l.83 : Narnaviruses have been previously identified in protists (Oomycetes and Leishmania).

Included “other protists” in the sentence so we can properly include were narnaviruses have been previously identified.

l.141: Indicate the parameters of ORF Finder (which genetic codes have been used for example). it can be critical when searching for protist viruses related to Lenarviricota as some could be mitochondria-associated.

Parameters of ORF Finder were added to the methods section.

l.161 : I would not use the the term "infection" but rather "presence", "association". As detailed in the Discussion part, bioinfo-based virus identification do not formally demonstrate host infection...

We changed the wording to better describe the association between the parasite and virus. It is true that we can not confirm that the virus is “infecting” the parasite, and we have revised the manuscript to reflect this.

l.184 : What is the level of ID shared between the second segments of MaRNAV1 and 2 ? S2 table mention only one percentage for both MaRNAV1 and 2... No additional segments could be found for MaRNAV4 ?

S2 table was updated to include the amino acid percent identities to the second segments of both MaRNAV1 and V2, and we also included an alignment file in fasta format of all the segments associated with MaRNA viruses. A sentence was also added to l.198 stating that no presence of the second segments was found in the samples of MaRNAV4.

l.197 : Replace "confirm" by "strongly suggest" ?

Done.

l.252 : The study in Charon et al has screened several SRA files from P. vivax Bioprojects to conclude that MaRNAV1 is widespread in P. vivax samples worldwide (not only in Malaysian samples).

We changed the sentence to focus only on the samples for MaRNAV2 that are from twelve Australian birds.

Table1 : Percent of identity with what ?

Specified what the percent identities are referring too.

Figure1: The quality is bad. Is the tree rooted ? What is the scale at the bottom ?

The tree is unrooted, with the scale being number of nucleotide substitutions per codon site. The quality of the image was also enhanced from 144x144 to 300 x 300. We have added the clarifying information to the figure legend “The scale refers to the number of nucleotide substitutions per codon site, and the tree is unrooted although the taxon Grapevine associated narnavirus is drawn at the root”.

Supplemental material: I would suggest sharing the alignment in fasta format. Could you share the Phyre2 info tables as well ?

Alignment file in fasta format included under S1Appendix/Matryoshka_virus_alignment. We also added the Phyre2 info tables to S1Appendix/Phyre2_pdb_files.

**Reviewer #2:** This is an interesting report. Perhaps the discussion is a little speculative, but I understand the need of providing a general context of otherwise a descriptive study. Some minor changes

Thank you for your comments and we hope to have addressed all of your concerns.

The phrase: “Even though protists are no less evolved than more complex multicellular eukaryotes, they play an important role in deducing the origins of RNA viruses today”

I understand the intention, but even mentioning a “scala naturae” to dismiss it without arguing against do not assist the reader without background in evolutionary biology. This is not the place to discuss it, but it is not simply a stylistic matter so I found such a phrase a little unsettling. The issue could be addressed by simply stating that the most recent common ancestor between protists and multicellular eukaryotes likely was part of the origin of eukaryotes and as such, study the RNA viruses in the extant lineages of protist and multicellular eukaryotes may inform about the common ancestor of the RNA viruses observed today.

Thank you for this comment and we agree that the phrase was unsettling. We have changed the wording of the sentence in accordance to your recommendation.

Lines between 255-275 are too speculative, I suggest changing it by emphasizing, briefly, the need of experimental work to establish the viruses effect, if any, in their hosts (in this case the Haemosporida parasites).

Thank you for the suggestion we have added a sentence on l. 271 of the original manuscript to address this issue.
